# Effect of Different Substitutions at the 1,7-Bay Positions of Perylenediimide Dyes on Their Optical and Laser Properties

**DOI:** 10.3390/molecules28196776

**Published:** 2023-09-23

**Authors:** Nathalie Zink-Lorre, Manuel G. Ramírez, Sara Pla, Pedro G. Boj, José A. Quintana, José M. Villalvilla, Ángela Sastre-Santos, Fernando Fernández-Lázaro, María A. Díaz-García

**Affiliations:** 1 Área de Química Orgánica, Instituto de Bioingeniería, Universidad Miguel Hernández de Elche, 03202 Elche, Spain; 2 Instituto Universitario de Física Aplicada a las Ciencias y Tecnologías, Universidad de Alicante, 03080 Alicante, Spain; ramirez@mscloud.ua.es; 3 Departamento de Óptica, Farmacología y Anatomía, and Instituto Universitario de Materiales (IUMA), Universidad de Alicante, 03080 Alicante, Spain; 4 Departamento de Física Aplicada and Instituto Universitario de Materiales (IUMA), Universidad de Alicante, 03080 Alicante, Spain

**Keywords:** perylenediimides, amplified spontaneous emission, photoluminescence, organic lasers

## Abstract

Perylenediimide (PDI) compounds are widely used as the active units of thin-film organic lasers. Lately, PDIs bearing two sterically hindering diphenylphenoxy groups at the 1,7-bay positions have received attention because they provide a way to red-shift the emission with respect to bay-unsubstituted PDIs, while maintaining a good amplified spontaneous emission (ASE) performance at high doping rates. Here, we report the synthesis of a series of six PDI derivatives with different aryloxy groups (**PDI 6** to **PDI 10**) or ethoxy groups (**PDI 11**) at the 1,7 positions of the PDI core, together with a complete characterization of their optical properties, including absorption, photoluminescence, and ASE. We aim to stablish structure-property relationships that help designing compounds with optimized ASE performance. Film experiments were accomplished at low PDI concentrations in the film, to resemble the isolated molecule behaviour, and at a range of increasing doping rates, to investigate concentration quenching effects. Compounds **PDI 10** and **PDI 7**, bearing substituents in the 2′ positions of the benzene ring (the one contiguous to the linking oxygen atom) attached to the 1,7 positions of the PDI core, have shown a better threshold performance, which is attributed to conformational (steric) effects. Films containing **PDI 11** show dual ASE.

## 1. Introduction

Optically pumped lasers based on solution-processed thin-film gain media are highly interesting as versatile, mechanically flexible, wavelength tunable active photonic devices with low-cost fabrication techniques that can fit any substrate and are useful for optical communications, sensing, and spectroscopy [1,2,3,4,5,6,7,8,9,10,11,12,13,14]. Among different options, dye-doped polymers are an attractive strategy to generate compact, flexible, and easy-to-handle solid-state organic lasers, as the small molecule active dye can be fine-tuned to fulfill all the physical requirements until reaching its best, and then the most appropriate polymeric matrix [e.g., polystyrene, PS, or poly(methyl methacrylate), PMMA] can be selected and the mixture can be optimized to achieve the desired material performances [15,16,17].

A key piece for a high laser performance is the active organic material, as it must generate high-quality photostable films with high gain and photoluminescence quantum yield (PLQY), and low waveguide losses. Thus, for example, aromatic compounds are especially well suited for laser applications because of the large oscillator strength of electronic transitions associated with the excitation of π-electrons. In this regard, perylenediimides (PDIs) have attracted great attention as dye lasers [16,18,19,20] due to their robustness against heat and chemicals (acids, bases, and oxidants), their strong absorption of visible light, and their high PLQY. Moreover, they are remarkably versatile compounds as their properties (solubility, absorption/emission maxima, redox potentials) can be modulated over a wide range by introduction of appropriate substituents on the different available positions of the PDI skeleton [21,22,23].

**Figure 1:**
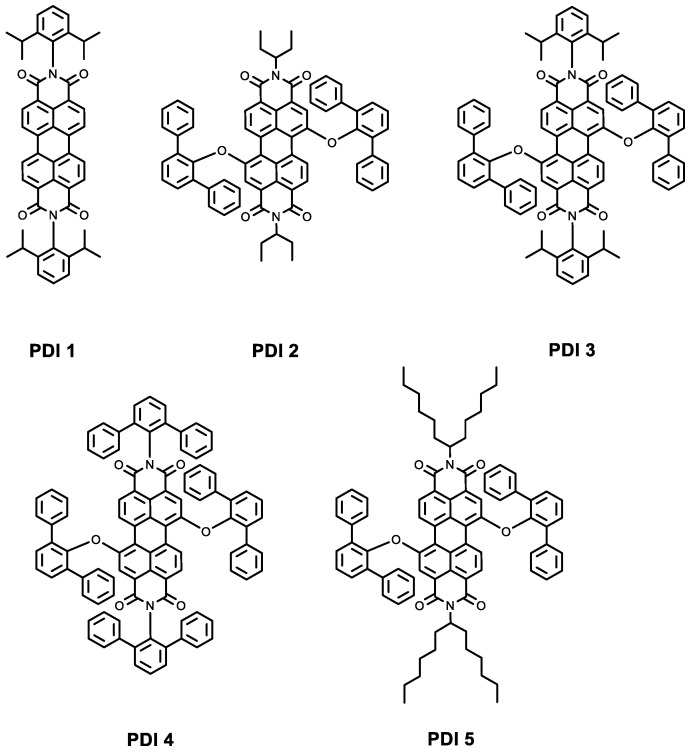
Structures of **PDI 1**–**PDI 5**.

Initially, PDIs without substituents at the bay positions (u-PDIs, bay-unsubstituted) were used for lasing [24] due to their superior performances on amplified spontaneous emission (ASE) generation [25,26,27,28], especially regarding photostability issues, being the best results in terms of energy threshold obtained for *N*,*N′*-bis(2,6-diisopropylphenyl)PDI (**PDI 1**, Figure 1) dispersed in a PS matrix at 1 wt% (with a 2−3 kW cm^−2^ ASE threshold) [25]. However, these compounds show some drawbacks, namely: (a) low dye loads in the polymer matrix (0.5–1 wt% of u-PDI with respect to PS) to avoid concentration-induced photoluminescence (PL) quenching and, therefore, a reduced emission efficiency; (b) their ASE thresholds are significantly larger than those of non-diluted molecular materials or semiconducting polymers; (c) absorption and PL spectra of u-PDIs are almost independent on the type of substituent attached to the N atoms because of the nodes on the HOMO and LUMO at the imide nitrogen atoms [16], so in all u-PDI-doped polymer films ASE appears at the same wavelength (ca. 579 nm) [25,26].

Attempts to design PDIs emitting at longer wavelengths led to investigate bay-substituted PDIs (b-PDIs), but they showed low ASE performances, mainly due to their distorted PDI cores resulting in low PLQY values [26]. However, an unexpected twist occurred when a 1,7-bis(diphenylphenoxy)PDI, **PDI 2** (Figure 1), with an undistorted core and, thus, a PLQY in liquid solution close to unity, appeared. PS films doped with this b-PDI displayed efficient ASE emission at wavelengths between 610 and 630 nm for a very wide range of concentrations. Indeed, thanks to the bulky groups attached, the maximum concentration was around 40 times larger (27 wt% of PDI with respect to PS) than typical PDI concentrations used in prior laser studies. This huge increase in the PDI load led to very high PL and ASE efficiencies, as well as low ASE thresholds [18].

This distinctive feature of **PDI 2** of enabling high loading rates in the hosting matrix without PL quenching was further exploited to investigate the role of the matrix. Thus, a detailed concentration dependence ASE study of a compound similar to **PDI 2** but with a higher solubility (provided by longer aliphatic chains at the imide positions), was carried out in both PMMA and PS, at even larger dye concentrations (i.e., 50 wt%) [16]. Further studies focused on the effect of modifying the group at the imide position in 1,7-bis(diphenylphenoxy)-substituted PDIs (compounds **PDI 3**–**PDI 5**, see Figure 1), aiming to optimize both threshold and photostability [29]. PDIs with aromatic substituents at the imide positions (**PID 3** and **PDI 4**) lasted ca. three times more than those bearing aliphatic chains (**PDI 5**), when prepared as diluted films without molecular aggregation, whilst as the PDI concentration increased, these differences tended to vanish. On the other hand, the use of aliphatic chains at the imide positions (**PDI 5**) led to lower ASE thresholds, as they are more effective in preventing molecular aggregation than the aromatic substituents [29].

An aspect of interest in the field of light emitting materials is the possibility to emit simultaneously at different colors. A possible approach towards this goal is to use compounds displaying dual ASE. Most of the examples reported in the literature correspond to materials in liquid solution, such as coumarins [30,31] or conjugated polymers, polyfluorene (PFO) being the most common one [32,33]. Fewer cases of dual ASE from solid films have been reported, most of them from PFO, in which the dual ASE is attributed to the coexistence of two phases of the polymer, which can emit simultaneously [34,35], or to the coexistence of a monomer and an excimer [36]. Aside from these, dual ASE was found in a bifluorene derivative single crystal, which was related to the emission from two different vibronic replicas [37]. More recently, dual ASE has been observed in large size nanographenes [38]. Here, dual ASE was attributed to the existence of losses, due to excited state absorption in the spectral region of the PL 0–1 transition at which ASE normally occurs. As a result, the ASE threshold for emission at the PL 0–0 band, generally large because of strong reabsorption, becomes like the one with emission at the PL 0–1 Consequently, ASE emission at both is observed simultaneously.

**Figure 2:**
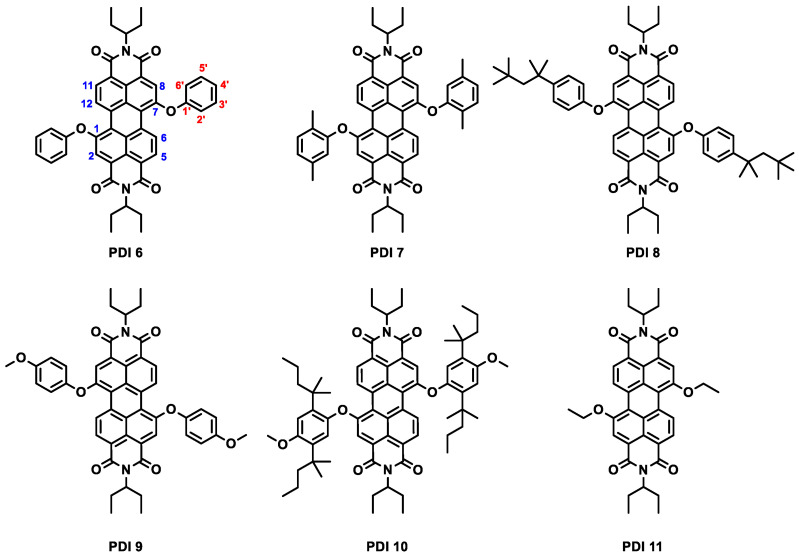
Structures of **PDI 6**–**PDI 11**. Blue numbers in **PDI 6** indicate the position of the substituents in the PDI core and red numbers the position of the substituents in the benzene ring.

In this paper, we continue our exploration of the optical and ASE properties of b-PDIs, in this case focusing on the effect of different substitutions on the bay positions of the PDI molecule. With this aim in mind, a series of PDI functionalized with different aryloxy or ethoxy groups in the bay positions have been synthesized (**PDI 6** to **PDI 11**, Figure 2). PS films containing these compounds have been prepared and their optical and ASE properties investigated. The effect of the PDI concentration in the film has also been explored. ASE has been observed in all compounds at different wavelengths, depending on the compound, and with different concentration-dependence behavior. Remarkably, dual ASE is observed for one of the compounds (**PDI 11**).

## 2. Results and Discussion

### 2.1. Preparation of Perylenediimide Derivatives

**Scheme 1:**
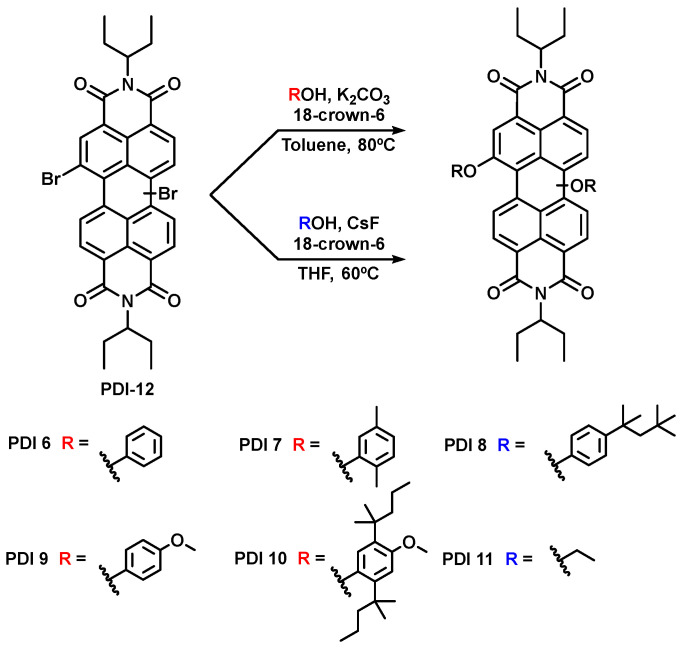
Synthetic routes to **PDI 6**–**PDI 11**.

The compound *N*,*N′*-di(ethylpropyl)-1,7(6)-dibromoperylenediimide, **PDI 12** (see chemical structure in Scheme 1), has been prepared following the described procedure [39], and selected as starting material for our study because of its high solubility in most organic solvents and for the ease with which it can be structurally modified giving rise to new derivatives.

**PDI 12** has been functionalized with different aromatic and aliphatic alcohols to obtain a family of compounds with the aim of studying and comparing them as active units in optical waveguides.

We synthesized the new compounds **PDI 6**, **PDI 7**, **PDI 9**, and **PDI 10** using the well precedented nucleophilic aromatic substitution of halogenated PDIs in basic media [21,40,41,42,43]. On the other hand, **PDI 8** and **PDI 11** were prepared using a fluoride-assisted substitution developed by us [44] (Scheme 1). The derivatives are obtained with good to very good yields as a mixture of 1,6 and 1,7 regioisomers (on average, the content of isomer 1,6 is ca. 22% as determined by ^1^H-NMR).

Among the prepared PDIs, **PDI 6** and **PDI 11** show the simplest structures, bearing phenoxy and ethoxy groups, respectively, at the 1,7 bay positions of the PDI core. The other compounds have a chemical structure, like that of **PDI 6**, but with additional substituents on different positions of the benzene rings, that increase the steric demand of the aromatic group (**PDI 7** and **PDI 8**), its electron density (**PDI 9**), or both (**PDI 10**). Interestingly, while the methyl groups located contiguous to the oxygen atoms in **PDI 7** may be responsible for intramolecular interactions, the more voluminous *tert*-octyl groups in *para* positions in **PDI 8** could only generate intermolecular interactions. On the other hand, the substituents in **PDI 10** may originate both intra- and intermolecular interactions.

The structure of all compounds was confirmed by ^1^H-NMR, ^13^C-NMR, UV-vis, IR, and MALDI-TOF MS (see Supplementary Materials).

Thermogravimetric analysis of the new compounds has been carried out to determine their stability, as done with other materials [45] (see Supplementary Materials). A very huge thermal stability till almost 400 °C is observed in all the PDIs, except for **PDI 7,** which starts losing weight at 65 °C, probably due to the elimination of the methylphenoxy substituents. All the PDIs melt between 130 °C and 250 °C, except **PDI 11,** whose melting point is above 300 °C (see Section 3).

### 2.2. Optical Properties of PDIs in Solution

All the synthesized compounds show strong optical absorption in the visible region of the solar spectrum, an expected characteristic of PDI-based materials (Table 1).

### 2.3. Optical Properties of PDIs in PS Thin Films

#### 2.3.1. Properties of Highly Diluted Films

**Figure 3:**
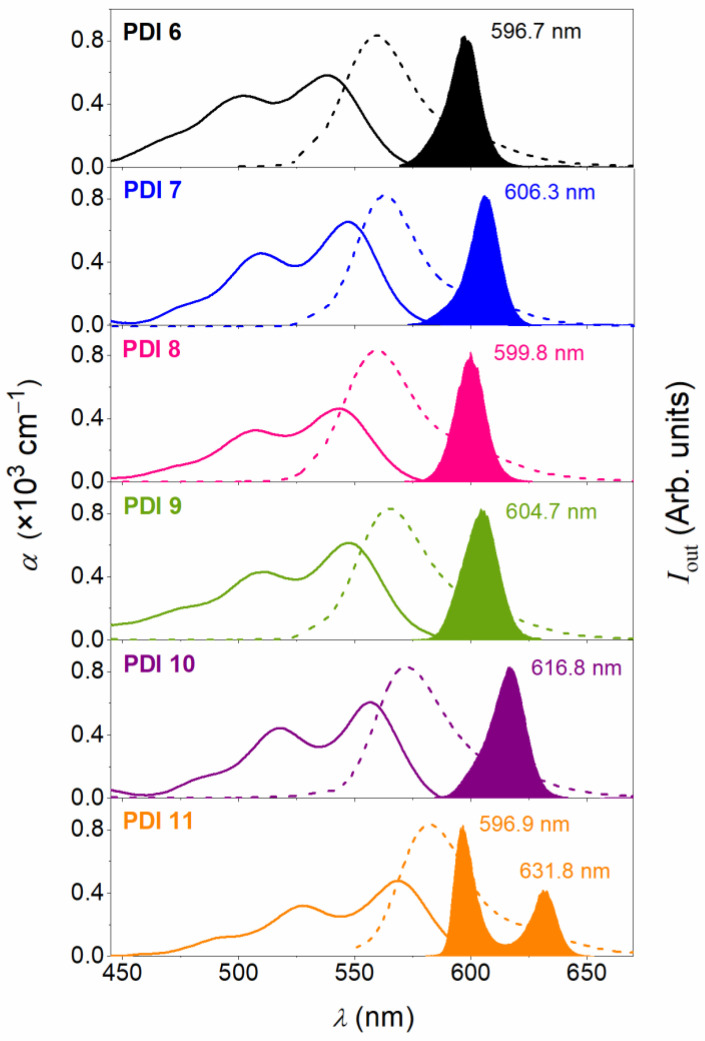
Optical properties of PS films doped with **PDI 6** to **PDI 11** (from top to down in panel). Dye content is around 5 μmol PDI/g PS (exact values in Table 2). Absorption coefficient, α (solid line, left axis), photoluminescence intensity (dashed line, right axis), and amplified spontaneous emission, ASE, intensity (filled area, right axis), versus wavelength, λ.

The absorption (ABS) and PL spectra for films containing a PDI compound, among the ones synthesized (**PDI 6** to **PDI 11**), dispersed at the lowest prepared concentration (around 5 μmol PDI/g PS), are shown in Figure 3. All relevant parameters are listed in Table 2. The ABS spectra for the various films have a similar shape, but their peaks appear at different wavelengths depending on the substituent at the bay positions (see Table 2). Particularly, **PDI 6** is the one whose main ABS peak occurs at a lower wavelength (538 nm). When the benzene group in the 1,7 bay positions of the PDI core is replaced by an aliphatic chain (as in **PDI 11**), a significant shift of 31 nm is observed (main ABS peak for **PDI 11** at 569 nm) because of the higher electron donor character of the alkoxy groups compared to the aromatic ones.

Focussing now on derivatives **PDI 7** to **PDI 10**, the presence of electron-donor alkyl and alkoxy substituents in the benzene groups attached to the bay-positions of the PDI core imply red-shifts of a few nm in the ABS peaks with respect to **PDI 6**: between 5 and 9 nm for **PDI 7**, **PID 8**, and **PDI 9**; and up to 19 nm for **PDI 10**. 

With regards to the PL spectra, the shapes for the various PDIs investigated are similar. Following the same trend observed in the absorption, the PL spectra for **PDI 7** to **PDI 10** are red shifted with respect to that of **PDI 6**.

The capacity of a given material to serve as active medium for a waveguide-based laser can be explored by studying its ASE properties, under pulsed excitation, when deposited as a thin waveguide film. The existence of ASE is evidenced by a narrowing of the PL spectrum at a certain pump intensity (called the ASE threshold), and simultaneously, an abrupt increase of the emission intensity [1,2,3,4]. The observation of ASE is indicative of the existence of gain due to the dominant contribution of stimulated emission over the spontaneous one. The ASE spectra for the prepared PDI-doped PS films are also shown in Figure 3. In all cases, a net narrowing of the PL emission is seen, with ASE peaks (λ_ASE_) at different wavelengths. The linewidth of the ASE emission is defined as the full width at half maximum (FWHM) and is typically of several nm (see exact values in Table 2). An interesting result is the possibility to tune λ_ASE_ over a relatively wide range (596–632 nm) by changing the type of substituents in the bay-positions of the PDI core.

The shifts in the λ_ASE_ values are a consequence of the shifts in the ABS and PL spectra as discussed above. These results open a way to tune the emission wavelength of waveguide-based lasers which include a laser resonator, for example, a diffractive grating, to conform the so-called distributed feedback (DFB) laser [1,2,3,4,46]. It should be noted that the DFB laser wavelength for a device built with an active film of a given thickness and active compound concentration depends on the geometrical parameters of the laser resonator (mainly the grating period). Nonetheless, the DFB laser threshold is optimized to its lowest possible value when its emission wavelength matches that at which ASE is observed. This is because ASE occurs at the wavelength at which the gain is maximal. In summary, the possibility to change the ASE wavelength of the active material, in this case by using different derivatives, provides a way to obtain optimized lasers (i.e., with lowest possible threshold) emitting at different wavelengths.

For **PDI 6** to **PDI 10**, ASE occurs very close to the first vibrational component of the PL spectrum (the 0–1), despite that it is less intense than the 0–0 component. In many materials investigated in the literature, ASE appears at the most intense vibronic transition. Very often this is the 0–1 transition, given that the intensity of the PL 0–0 component is reduced by self- or re-absorption due to the proximity between absorption and PL [3,4]. This is a very common situation in highly planar and stable derivatives with small Stokes shift, such as perylenediimides [18,25], or carbon-bridged oligophenylenevinylene, COPV*n* (with *n* = 1 to 6) [47]. Interestingly, in the film containing **PDI 11**, ASE is observed simultaneously at two different wavelengths (596.9 nm and 631.8 nm), close to the 0–0 and 0–1 PL transitions, respectively. According to the behavior observed in nanographenes, a possible explanation would be the existence of losses, for example, due to triplets or to excited absorption bands, in the spectral region of the 0–1 PL transition, at which ASE normally occurs. To ascertain the particular underlying mechanisms, further photophysical studies, such as transient absorption spectroscopy, are required. These might be the subject of future studies.

**Figure 4:**
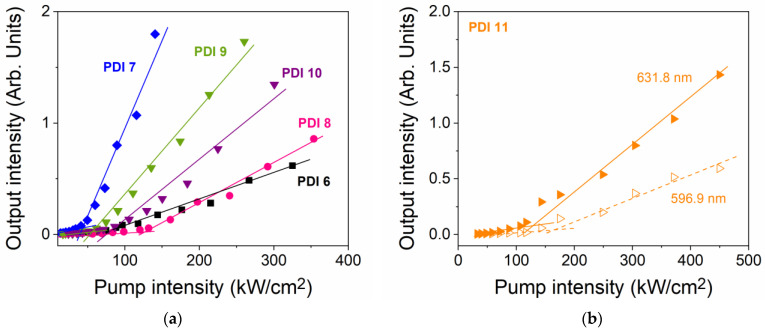
Output intensity versus pump intensity for ASE threshold determination for PS films doped with **PDI 6** to **PDI 10** (**a**), and **PDI 11** (**b**). Dye content is around 5 μmol PDI-*n*/g (exact values in Table 2). For **PDI 11**, curves for the two ASE peaks are shown (at 596.9 nm, empty triangles; and at 631.8 nm, filled triangles). Full and dashed lines are guides to the eye.

With regards to the ASE thresholds, they are determined from plots of the output intensity versus the pump intensity. Such plots for all the compounds are shown in Figure 4. Numerical values (listed in Table 2) correspond to the pump intensity at which a clear change of slope is observed. The lowest thresholds are obtained for **PDI 7**, **PDI 9**, and **PDI 10**, with values of 40, 50, and 62 kW cm^−2^, respectively. Larger values are obtained for **PDI 6** (87 kW cm^−2^) and **PDI 8** (127 kW cm^−2^) (see Figure 4a). The behavior of **PDI 11** is analyzed separately because it shows dual ASE (see Figure 4b). Plots for each of the two ASE peaks are represented and the corresponding thresholds obtained. These are not very different (167 and 129 kW cm^−2^, for the emissions at 596.9 and 631.8 nm, respectively), which justifies the observation of both simultaneously. Interestingly, despite that the energy is shared between two peaks, the thresholds are not very large (the one for the peak at 631.8 nm is like that of **PDI 8**).

#### 2.3.2. Dye-Concentration Dependence of the Properties

**Figure 5:**
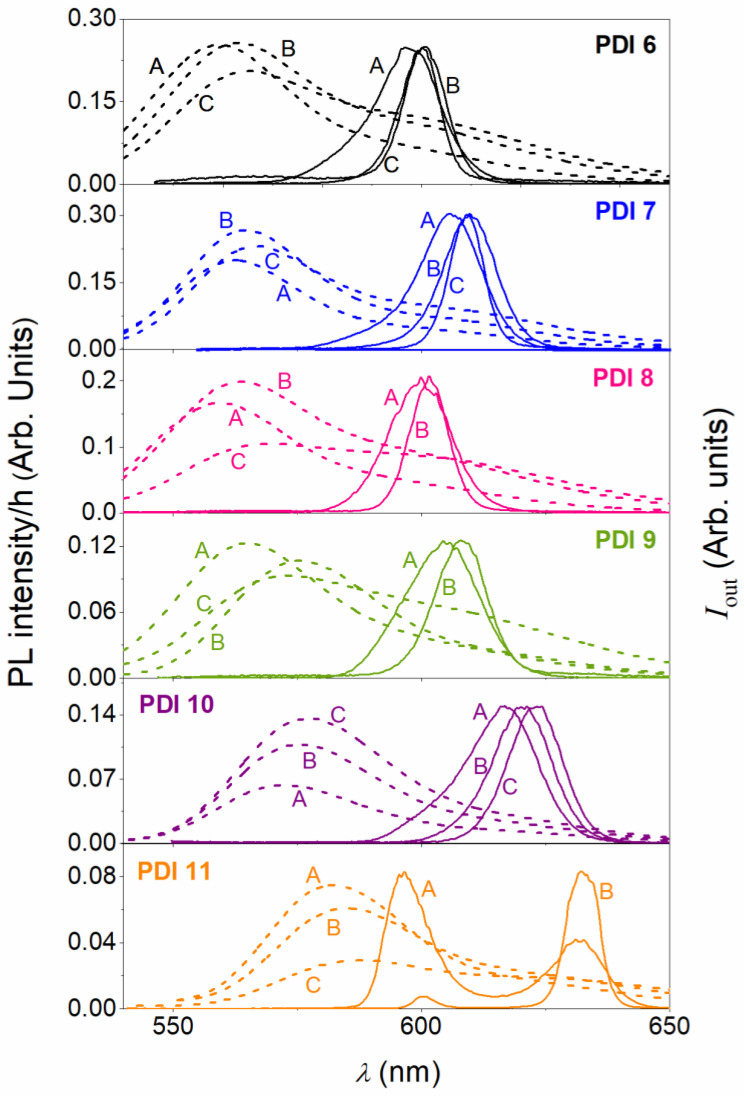
Effect of the PDI concentration in the PS film, on the PL and ASE spectra. PL spectra divided by film thickness (left axis) and ASE (right axis) for films containing **PDI 6** to **PDI 11** (from top to down in panel) at three different concentrations (in μmol PDI/g PS): 4.9 (label A), 14.9 (label B), and 30.2 (label C).

**Figure 6:**
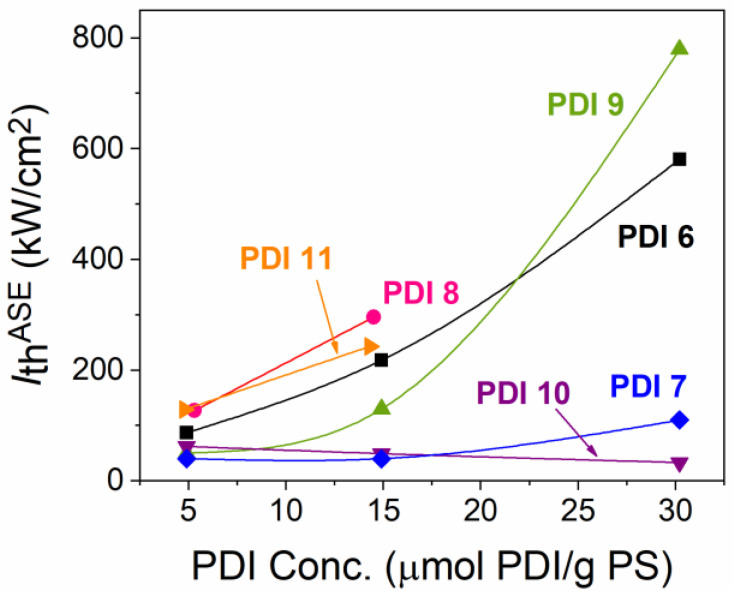
Evolution of the ASE threshold (*I*_th-ASE_) with the dye concentration in PS for the various compounds: **PDI 6** (black squares), **PDI 7** (blue diamonds), **PDI 8** (pink circles), **PDI 9** (green upside triangles), **PDI 10** (purple inverted triangles), **PDI 11** (orange twisted triangles). The lines are guides to the eye.

The differences among the studied compounds (**PDI 6** to **PDI 11**) become more pronounced when analysing the performance of dye-doped PS films at higher dye contents. Particularly, for each compound, besides the film doped with around 5 μmol PDI/g PS, discussed in Section 2.3.1, films with two additional concentrations were prepared (around 15 and 30 μmol PDI/g PS) and their properties analyzed. Figure 5 shows the PL and ASE spectra for all the prepared films. ASE thresholds, for all the cases in which ASE was observed, were obtained from output intensity versus pump intensity curves, such as the ones plotted in Figure 4. They have been represented as a function of the dye concentration in Figure 6. All numerical data have also been collected in Table 2.

Among all the studied compounds, the one that aggregates less, as the concentration increases, is **PDI 10**. This is the one with bulky substituents in the 2′ and 5′ positions of the benzene group attached to the 1,7 bay positions of the PDI core. The capacity to aggregate is inferred from the PL spectra (Figure 5) and from the concentration dependence of the ASE threshold (Figure 6). The PL spectrum keeps its shape as the concentration increases and its intensity grows proportionally, with just small red-shifts. Besides, ASE is observed for the three concentrations and the ASE peak red-shifts in accordance with the PL shifts. Moreover, a low ASE threshold is maintained for the three concentrations studied, getting its lowest value (33 kW cm^−2^) at the highest concentration (see Figure 6). The other compound that shows good behavior in this respect, although not as good as that of **PDI 10**, is **PDI 7**. For the latter, the PL spectrum keeps its shape, and its intensity grows proportionally, but only for the second concentration 14.9 μmol PDI/g PS. For the highest concentration, a certain saturation is observed which is indicative of aggregation or molecular interaction leading to PL quenching. Besides, although ASE appears, its wavelength is approximately the same as for the previous concentration. Also, in this case the ASE threshold is low for the three concentrations, with some increase (to 110 μmol PDI/g PS) for the highest one. The rest of compounds clearly aggregate more rapidly as the concentration increases. This is evidenced by changes in the PL spectra as well as a decrease of the PL intensity as the concentration increases. This is also supported by the concentration dependence of the ASE threshold (Figure 6). Particularly, at the highest concentration, the ASE thresholds of **PDI 6** and **PDI 9** become significantly higher (above 400 kW cm^−2^), while no ASE is observed for **PDI 8** and **PID 11**. For the latter compound, it should be noted that dual ASE appears only at the lowest concentration. When the concentration is increased to 15 μmol PDI/g PS, a single ASE peak is observed (at around 632.7 nm), which is the only related to the PL 0–1 transition. This is attributed to the increase of reabsorption losses at the other peak, as a consequence of the increase of the dye concentration.

The better performance of compounds **PDI 10** and **PDI 7** might be attributed to conformational (steric) effects, due to the substitutions in the different positions of the benzene group attached to the 1,7 positions. Studying the NMR spectra of **PDI 6**–**PDI 10** (see Supplementary Materials), it is easy to assign the doublets lying downfield to the protons of positions 6 and 12 of the perylene core, i.e., the protons in front of the oxygen atoms. The signals appear at 9.62, 9.71, 9.58, 9.64, and 9.77 ppm for **PDI 6** to **PDI 10**, respectively. These chemical shifts may be related to the strength of the hydrogen bond interaction between the oxygen and the H-6/H-12 atoms. The most deshielded doublets correspond to **PDI 10** and **PDI 7**, in this order, indicating a stronger intramolecular hydrogen bond interaction and, consequently, a more rigid structure. This may be a consequence of the steric hindrance introduced by the substituents located in the 2′ position of the benzene ring, i.e., the position contiguous to the oxygen atom, which hinders the free rotation of this ring.

It is interesting to compare the obtained ASE threshold values to those of previously reported bay-substituted PDIs (i.e., **PDI 3** and **PDI 5**) [29]. At a molar concentration of around 30 μmol PDI/g PS these compounds showed thresholds of around 50 and 20 kW cm^−2^, respectively. The value obtained for **PDI 10** (33 kW cm^−2^) is between both.

The operational ASE lifetime has been studied for the film with the lowest threshold (**PDI 10** at the 30.2 μmol PDI/g PS), under pump at both, moderate conditions (two times above the threshold), and extreme conditions (around 2500 kW cm^−2^). For that purpose, the ASE intensity, recorded at the same spot of the sample under continuous pump, has been represented as a function of time (or the number of pump pulses); see Figure S31 in the supporting information. This property has been quantified through the so-called ASE half-life (τ_1/2_^ASE^), defined as the time or the number of pump pulses at which the ASE intensity decays to half of its initial value. ASE half-lives for **PDI 10**, at the 30.2 μmol PDI/g PS concentration, were around 2.8 × 10^3^ and 0.8 × 10^3^ pump pulses, for moderate and extreme pumping conditions, respectively. The latter value is somewhat lower than those previously reported for films containing **PDI 3** and **PDI 5**, at similar molar concentrations and measured at the same excitation conditions (2500 kW cm^−2^): 8 × 10^3^ and 3 × 10^3^ pump pulses, respectively.

## 3. Materials and Methods

### 3.1. Chemicals and Instruments

Solvents and reagents were obtained from commercial sources and used as received. PS (M = 35,000 g mol^−1^) was purchased from Merk KGaA (Darmstadt, Germany) Column chromatography: SiO_2_ (40–63 μm). TLC plates coated with SiO_2_ 60F254 were visualized by UV light. NMR spectra (300 MHz for ^1^H, 75 MHz for ^13^C) were recorded at 25 °C using a Bruker AC300 spectrometer (Bruker Corporation, Billerica, MA, USA). The solvents for spectroscopic studies were of spectroscopic grade and used as received. UV-vis spectra in solution were measured with a Helios Gamma spectrophotometer (Thermo Spectronic, Cambridge, UK). Fluorescence emission spectra in solution were recorded on a Perkin Elmer LS55 spectrofluorometer (PerkinElmer, Inc., Waltham, MA, USA) and fluorescence quantum yields were calculated using *N*,*N′*-di(hexylheptyl)perylenediimide as the standard (Φ_f_ = 1) [21]. IR spectra were recorded in KBr pellets with a Nicolet Impact 400D spectrophotometer (Thermo Electron Corporation, Madison, WI, USA). High resolution Mass spectra were obtained from a Bruker Reflex II matrix-assisted laser desorption/ionization time of flight (MALDI-ToF) (Bruker Corporation, Billerica, MA, USA) using dithranol as matrix. Melting points were measured in a Stuart Scientific melting point apparatus smp3. Thermogravimetric analyzes were measured in a TG-DSC2 METTLER-TOLEDO equipment.

### 3.2. Synthesis of Perylenediimide Derivatives ***PDI 6**–**PDI 11***

***N,N*** ***′*-Di(ethylpropyl)-1,7(6)-diphenoxy-3,4:9,10-perylenetetracarboxydiimide (PDI 6)**

A mixture of phenol (151 mg, 1.6 mmol), K_2_CO_3_ (402 mg, 2.9 mmol), and 18-crown-6 (1.57 g, 5.90 mmol) was stirred in dry toluene (50 mL) at room temperature under argon for 20 min. Then, *N*,*N′*-diethylpropyl-1,7(6)-dibromoperylene-3,4:9,10-tetracarboxidiimide, **PDI 12**, (264 mg, 0.38 mmol) was added and the reaction mixture was stirred for 5 h at 80 °C under argon atmosphere. After cooling to room temperature, the solvent was distilled off and the residue was chromatographed (SiO_2_, dichloromethane) to afford the regioisomeric mixture (1,6:1,7 22:78) of **PDI 6** (230 mg, 84%) as a red solid. Mp: 245 °C.

^1^H NMR (300 MHz, CD_2_Cl_4_): *δ* = 9.62 (dd, 2H, *J* = 8.4 Hz, 2x*H*-PDI), 8.62 (dd, 2H, *J =* 8.4 Hz, 2x*H*-PDI), 8.33 (s, 2H, *H*-PDI), 7.52 (m, 4H, 2x*H*-Ph), 7.25 (m, 6H, 2x*H*-Ph), 4.99 (m, 2H, 2xN-C*H*(CH_2_-CH_3_)_2_), 2.18 (m, 4H, 2xN-CH(C*H*_2_-CH_3_)_2_), 1.94 (m, 4H, 2xN-CH(C*H*_2_-CH_3_)_2_), 0.93 (t, 12H, 2xN-CH(CH_2_-C*H*_3_)_2_) ppm; ^13^C NMR (75 MHz, CD_2_Cl_4_, 60 °C): *δ*. 164.11, 156.51, 155.46, 155.28, 155.16, 133.54, 131.31, 130.94, 129.50, 129.08, 128.03, 125.62, 125.56, 125.51, 124.15, 124.04, 122.00, 120.09, 119.96, 58.20, 57.96, 57.73, 25.27, 11.77 ppm; IR (KBr) *ν*: 2950, 2932, 2868, 1701 (C=O imide), 1654 (C=O imide), 1596, 1485, 1409, 1328, 1258, 1211, 1047, 925, 803, 768, 692 cm^−1^; UV-vis (chloroform), *λ*_max_ (log *ε*): 404 (3.8), 510 (4.4) 542 nm (4.6); MS (MALDI-TOF) *m*/*z*: calcd for C_46_H_38_N_2_O_6_ 714.2724 [*M*]^−^, found 714.2244 [*M*]^−^.

***N*,*N*** ***′*-Di(ethylpropyl)-1,7(6)-di(2,5-dimethylphenoxy)-3,4:9,10-perylenetetracarboxydiimide (PDI 7)**

A mixture of 2,5-dimethylphenol (109 mg, 0.89 mmol), K_2_CO_3_ (170 mg, 1.23 mmol), and 18-crown-6 (875 mg, 3.31 mmol) was stirred in dry toluene (40 mL) at room temperature under argon for 20 min. Then, **PDI 12** (100 mg, 0.15 mmol) was added and the reaction mixture was stirred for 5 h at 80 °C under argon atmosphere. After cooling to room temperature, the solvent was distilled off and the residue was chromatographed (SiO_2_, chloroform) to afford the regioisomeric mixture (1,6:1,7 25:75) of **PDI 7** (103 mg, 84%) as a red solid. Mp: 145 °C.

^1^H NMR (300 MHz, CDCl_3_): *δ* = 9.70 (d, 2H, *J* = 8.4 Hz, 2x*H*-PDI), 8.60 (d, 2H, *J* = 8.4 Hz, 2x*H*-PDI), 8.17 (s, 2H, 2x*H*-PDI), 7.28 (d, 4H, *J* = 7.4 Hz, 2x*H*-Ph), 7.05 (d, 4H, *J* = 7.5 Hz, 2x*H*-Ph), 6.88 (s, 2H, 2x*H*-Ph), 5.08–4.92 (m, 2H, 2xN-C*H*(CH_2_-CH_3_)_2_), 2.33 (s, 6H, 2xPh-C*H*_3_), 2.28 (s, 6H, 2xPh-C*H*_3_), 2.24–2.12 (m, 4H, 2xN-CH(C*H*_2_-CH_3_)_2_), 1.98–1.80 (m, 4H, 2xN-CH(C*H*_2_-CH_3_)_2_), 0.89 (t, 12H, *J* = 7.5 Hz, 2xN-CH(CH_2_-C*H*_3_)_2_) ppm; ^13^C NMR (75 MHz, CD_2_Cl_4_, 60 ºC): *δ* 166.9, 166.6, 159.8, 158.7, 155.4, 155.3, 141.1, 136.6, 136.4, 134.9, 134.1, 132.8, 132.3, 131.9, 131.6, 131.1, 130.5, 129.8, 129.7, 129.6, 129.5, 127.8, 126.9, 126.8, 125.5, 125.2, 124.7, 123.9, 123.8, 123.3, 60.6, 32.5, 27.9, 23.9, 18.7, 14.3 ppm; IR (KBr) *ν*: 2958, 2917, 2872, 1699 (C=O imide), 1650 (C=O imide), 1593, 1503, 1405, 1327, 1262, 1200, 1111, 1053, 804, 751 cm^−1^; UV-vis (chloroform), *λ*_max_ (log *ε*): 398 (3.89), 508 (4.53), 546 (4.71) nm; MS (MALDI-TOF) *m*/*z*: calcd for C_50_H_46_N_2_O_6_ 770.3356 [*M*]^−^, found 770.3111 [*M*]^−^.

***N*,*N′*-Di(ethylpropyl)-1,7(6)-di(4-*t*-octylphenoxy)-3,4:9,10-perylenetetracarboxydiimide (PDI 8)**

A mixture of 4-*t*-octylphenol (119 mg, 0.58 mmol), CsF (106 mg, 0.7 mmol), and 18-crown-6 (739 mg, 2.8 mmol) was added to a solution of 100 mg (0.14 mmol) of **PDI 12** in dry THF (2 mL). The reaction was refluxed 6 h under argon atmosphere and, after cooling, it was extracted with dichloromethane and washed with water. The organic layer was dried over anhydrous sodium sulfate, filtered, and evaporated. Purification was carried out by silica gel column chromatography using CH_2_Cl_2_:hexane 1:1 as eluent yielding 128 mg (98%) of **PDI 8** as a regioisomeric mixture (1,6:1,7 27:73) of a red powder. Mp: 131 °C.

^1^H NMR (300 MHz, CDCl_3_): *δ* = 9.56 (dd, 2H, *J =* 8.4 Hz, 2x*H*-PDI), 8.59 (dd, 2H, *J =* 8.4 Hz, 2x*H*-PDI), 8.33 (s, 1H, *H*-PDI), 8.25 (s, 1H, *H*-PDI),7.46 (d, 4H, *J =* 8.9 Hz, 2x*H*-Ph), 7.09 (d, 4H, *J =* 8.8 Hz, 2x*H*-Ph), 5.00 (m, 2H, 2xN- C*H*(CH_2_-CH_3_)_2_), 2.18 (m, 4H, 2xN-CH(C*H*_2_-CH_3_)_2_), 1.90 (m, 4H, 2xN-CH(C*H*_2_-CH_3_)_2_), 1,76 (br, 4H, 2xPh-C(CH_3_)_2_-C*H*_2_-C(CH_3_)_3_), 1.42 (br, 12H, 2xPh-C(C*H*_3_)_2_-CH_2_-C(CH_3_)_3_), 0.89 (m, 12H, 2xN-CH(CH_2_-C*H*_3_)_2_), 0.79 (m, 18H, 2xPh-C(CH_3_)_2_-CH_2_-C(C*H*_3_)_3_) ppm; ^13^C NMR (75 MHz, CDCl_3_): *δ* 164.25, 163.77, 156.46, 155.37, 152.43, 152.32, 147.34, 147.22, 133.32, 133.24, 130.12, 129.21, 128.74, 128.19, 128.17, 127.66, 125.20, 123.87, 123.63, 122.34, 122.16, 118.91, 118.77, 57.77, 57.60, 57.42, 57.14, 38.44, 38.43, 38.42, 32.42, 32.41, 32.41, 31.81, 31.44, 29.67, 24.95, 22.66, 14.09, 11.32 ppm;. IR (KBr) *ν*: 2961, 2880, 1701 (C=O imide), 1666 (C=O imide), 1596, 1497, 1409, 1328, 1258, 1199, 1170, 1053, 1007, 925, 803, 756 cm^−1^; UV-vis (chloroform) *λ*_max_ (log *ε*): 407 (4.0), 511 (4.5), 545 (4.7) nm; MS (MALDI-TOF) *m*/*z*: calcd for C_62_H_70_N_2_O_6_ 938.5228 [*M*]^−^, found 938.5149 [*M*]^−^.

***N*,*N′*-Di(ethylpropyl)-1,7(6)-di(4-methoxiphenoxy)-3,4:9,10-perylenetetracarboxydiimide (PDI 9)**

A mixture of 4-methoxyphenol (151 mg, 1.22 mmol), K_2_CO_3_ (339 mg, 2.46 mmol), and 18-crown-6 (1.29 g, 4.90 mmol) was stirred in dry toluene (50 mL) at room temperature under argon for 20 min. Then, **PDI 12** (200 mg, 0.29 mmol) was added and the reaction mixture was stirred for 5 h at 80 °C under argon atmosphere. After cooling to room temperature, the solvent was distilled off and the residue was chromatographed (SiO_2_, dichloromethane) to afford the regioisomeric mixture (1,6:1,7 11:89) of **PDI 9** (152 mg, 67%) as a red solid. Mp: 243 °C.

^1^H NMR (300 MHz, CDCl_3_): *δ* = 9.64 (d, 2H, *J =* 8.4 Hz, 2x*H*-PDI), 8.60 (d, 2H, *J =* 8.3 Hz, 2x*H*-PDI), 8.29 (s, 2H, 2x*H*-PDI), 7.14 (d, 4H, *J =* 9.1 Hz, 4x*H*-Ph), 7.00 (d, 4H, *J =* 9.1 Hz, 4x*H*-Ph), 5.07–4.97 (m, 2H, 2xN-C*H*(CH_2_-CH_3_)_2_), 3.86 (s, 6H, 2xO-C*H*_3_), 2.26–2.14 (m, 4H, *J =* 7.3 Hz, 2xN-CH(C*H*_2_-CH_3_)_2_), 1.96–1.82 (m, 4H, *J =* 7.3 Hz, 2xN-CH(C*H*_2_-CH_3_)_2_), 0.88 (t, 12H, *J =* 7.4 Hz, 2xN-CH(CH_2_-C*H*_3_)_2_) ppm; ^13^C NMR (75 MHz, CDCl_3_): *δ* = 157.2, 157.1, 156.2, 148.2, 133.4, 129.3, 128.8, 125.0, 123.1, 121.3, 121.2, 115.7, 57.6, 55.7, 29.7, 25.0, 11.3 ppm; IR (KBr) *ν*: 2966, 2913, 2876, 1691 (C=O imide), 1654 (C=O imide), 1593, 1499, 1405, 1323, 1249, 1200, 1033, 923, 806, 767 cm^−1^; UV-vis (chloroform) *λ*_max_ (log *ε*): 408 (3.97), 514 (4.56), 550 (4.73) nm; MS (MALDI-TOF) *m*/*z*: calcd for C_48_H_42_N_2_O_6_ 774.2941 [*M*]^−^, found 774.2883 [*M*]^−^.

***N*,*N′*-Di(ethylpropyl)-1,7(6)-di[2,5-bis(1,1-dimethylbuthyl)-4-methylphenoxy]-3,4:9,10-perylenetetracarboxydiimide (PDI 10)**

A mixture of 2,5-bis(1,1-dimethylbuthyl)-4-methylphenol (1.60 g, 5.48 mmol), K_2_CO_3_ (1.61 g, 11.65 mmol), and 18-crown-6 (3.92 g, 14.96 mmol) was stirred in dry toluene (400 mL) at room temperature under argon for 20 min. Then, **PDI 12** (1.00 g, 1.46 mmol) was added and the reaction mixture was stirred for 5 h at 80 °C under argon atmosphere. After cooling to room temperature, the solvent was distilled off and the residue was chromatographed (SiO_2_, dichloromethane) to afford the regioisomeric mixture (1,6:1,7 24:76) of **PDI 10** (1.31 g, 81%) as a red solid. Mp: 216 °C.

^1^H NMR (300 MHz, CDCl_3_): *δ* = 9.77 (d, 2H, *J =* 8.8 Hz, 2x*H*-PDI), 8.57 (d, 2H, *J =* 8.4 Hz, 2x*H*-PDI), 8.15 (s, 2H, 2x*H*-PDI), 6.93 (s, 2H, 2x*H*-Ph), 6.77 (s, 2H, 2x*H*-Ph), 5.04–4.94 (m, 2H, 2xN-C*H*(CH_2_-CH_3_)_2_), 3.91 (s, 6H, 2xO-C*H*_3_), 2.34–2.10 (m, 4H, 2xN-CH(C*H*_2_-CH_3_)_2_), 1.94–1.79 (m, 4H, 2xN-CH(C*H*_2_-CH_3_)_2_), 1.68–1.11 (m, 40H, 2xPh-(C(C*H*_3_)_2_-(C*H*_2_)_2_-CH_3_)_2_), 0.98–0.85 (m, 24H, 2xN-CH(CH_2_-C*H*_3_)_2_.+ 2xPh-(C(CH_3_)_2_-(CH_2_)_2_-C*H*_3_)_2_) ppm; ^13^C NMR (75 MHz, CD_2_Cl_4_): *δ* = 164.1, 163.2, 158.0, 156.7, 155.5, 155.4, 146.2, 146.0, 138.3, 138.2, 136.8, 134.1, 133.7, 131.0, 129.5, 129.3, 127.9, 127.8, 127.7, 126.6, 124.7, 123.8, 123.6, 123.1, 122.8, 122.3, 122.0, 121.3, 121.1, 112.6, 58.2, 57.5, 55.6, 45.2, 43.4, 38.0, 37.7, 28.8, 28.6, 28.1, 28.0, 27.9, 25.0, 24.9, 18.4, 18.3, 18.2, 14.7, 14.6, 11.3, 11.1, 11.0 ppm; IR (KBr) *ν*: 2945, 2872, 1699 (C=O imide), 1662 (C=O imide), 1593, 1499, 1401, 1319, 1266, 1176, 1115, 1053, 812, 743 cm^−1^; UV-vis (chloroform) *λ*_max_ (log *ε*): 523 (4.45), 563 (4.62) nm; MS (MALDI-TOF) *m*/*z*: calcd for C_72_H_90_N_2_O_8_ 1110.6697 [*M*]^−^; found 1110.6758 [*M*]^−^.

***N*,*N′*-Di(ethylpropyl)-1,7(6)-diethoxy-3,4:9,10-perylenetetracarboxydiimide (PDI 11)**

A mixture of ethanol (30 μL, 0.58 mmol), CsF (106 mg, 0.7 mmol), and 18-crown-6 (739 mg, 2.8 mmol) was added to a solution of 100 mg (0.1 mmol) of **PDI 12** in dry THF (2 mL). The reaction was refluxed for 6 h under an argon atmosphere, and after cooling, it was extracted with dichloromethane and washed with water. The organic layer was dried over anhydrous sodium sulfate, filtered, and evaporated. Purification was carried out by silica gel column chromatography using CH_2_Cl_2_:hexane 1:1 as eluent yielding 83 mg (97%) of **PDI 11** as a regioisomeric mixture (1,6:1,7 23:77) of a purple powder. Mp: > 300 °C.

^1^H NMR (300 MHz, CDCl_3_) *δ* = 9.62 (d, 2H, *J =* 8.4 Hz, 2x*H*-PDI), 8.68–8.55 (dd, 2H, *J =* 8.3 Hz, 2x*H*-PDI), 8.48 (s, 1H, *H*-PDI), 8.37 (s, 1H, *H*-PDI), 5.07 (m, 2H, 2xN-C*H*(CH_2_-CH_3_)_2_), 4.58 (m, 4H, 2xO-C*H*_2_-CH_3_), 2.28 (m, 4H, *J =* 7.4 Hz, 2xN-CH(C*H*_2_-CH_3_)_2_), 1.94 (m, 4H, *J =* 7.4 Hz, 2xN-CH(C*H*_2_-CH_3_)_2_), 1.71 (t, 6H, *J =* 6.9 Hz, 2xO-CH_2_-C*H*_3_), 0.93 (t, 12H, 2xN-CH(CH_2_-C*H*_3_)_2_) ppm; ^13^C NMR (75 MHz, CDCl_3_): *δ* = 157.82, 156.66, 134.29, 133.83, 130.79, 129.30, 128.59, 127.98, 127.22, 123.88, 123.59, 121.50, 120.75, 117.70, 66.11, 57.60, 29.68, 25.05, 15.08, 11.32 ppm;. IR (KBr) *ν*: 2967, 2938, 2874, 1701 (C=O imide), 1660 (C=O imide), 1590, 1526, 1415, 1328, 1269, 1205, 1065, 1013, 808, 750 cm^−1^; UV-vis (chloroform) *λ*_max_ (log *ε*): 402 (3.8), 531 (4.5), 568 (4.7) nm; MS (MALDI-TOF) *m*/*z*: calcd for C_38_H_38_N_2_O_6_ 618.2724 [*M*]^−^, found 618.2609 [*M*]^−^.

### 3.3. Thin Film Fabrication

Thin films consisted of PS (used as a matrix), with a PDI derivative (among the ones investigated, **PDI 6** to **PDI 11**, see Figure 2) dispersed on it, deposited over quartz substrates. They were prepared by spin-coating with a Module LabSpin 618 apparatus (Süss Microtec SE, Garching, Germany), using toluene as a solvent. The PDI concentration with respect to PS was varied between 4.9 μmol PDI/g PS and 31.1 μmol PDI/g PS, which corresponds approximately to the range 0.3 and 3.3 wt% of PDI with respect to PS. For a proper comparison, films with the same molar concentration were used. Film thickness (*h*_f_) was adjusted through the concentration of total mass with respect to the solvent to obtain values of around 600 nm (measured with an interferometer coupled to an optical microscope). This election aims to obtain films that support fundamental transversal waveguide modes (TE_0_ and TM_0_) with a high confinement factor (Γ ≈ 90%), which is convenient to optimize the ASE thresholds. Films were annealed, after preparation, at 90 ºC for around 2 h in a Vaciotem-TV oven (J.P. Selecta, Abrera, Barcelona, Spain) to eliminate residual solvent.

### 3.4. Optical Characterization

Film absorption (ABS) and PL emission measurements were performed in a Jasco V-650 spectrophotometer (Tokyo, Japan) and a Jasco FP-6500/6600 fluorimeter (Tokyo, Japan), respectively. The absorption coefficient (*α*) was calculated through the expression *α* = ln(10)·*A*/*h*_f_.

The ASE characterization was performed under excitation with the second harmonic of a Nd:YAG laser (532 nm, 10 ns, 10 Hz), model Indi-40, provided by Spectra-Physiscs (MKS Instruments Inc., Andover, MA, USA), with a setup previously described [18,20]. The pump beam was expanded and collimated, and by means of a circular aperture, the central part was selected, so the beam intensity was approximately uniform. The energy of the pump pulses was controlled using neutral density filters. The beam was then shaped with a cylindrical lens into a stripe of 3.5 mm of width. The length of the stripe was adjusted to 0.5 mm by means of an adjustable slit. This stripe was projected perpendicularly to the film, so one of its edges coincided with one of the films edges, from which the PL light emitted was collected with an Ocean Optics USB2000-UV-VIS fibre spectrometer provided by Ocean Insight (Orlando, FL, USA) with 1.3 nm spectral resolution. The presence of ASE is evidenced by a narrowing of the emitted spectrum at a certain pump intensity (the ASE threshold, *I*_th_^ASE^). Simultaneously, a sudden increase of the output intensity (*I*_out_) is observed.

## 4. Conclusions

We have synthesized six PDI derivatives with different types of substituents at the 1,7 bay-positions of the PDI core, namely **PDI 6** to **PDI 10,** bearing aryloxy groups, and **PDI 11** ethoxy groups, and characterized their optical properties and their capacity to serve as active media for laser devices. To date, only 1,7-bis(diphenylphenoxy)-substituted PDIs had been used for lasing. Results show that the type of substituents attached to the 1,7 positions of the PDI core (phenoxy in **PDI 6**, versus ethoxy in **PDI 11**) and to the positions of the substituents in the benzene ring (in **PDI6** to **PDI 10**) significantly affects the absorption, PL, and ASE spectra. Indeed, λ_ASE_ covers a relatively wide range (596–632 nm), depending on the compound, which constitutes an interesting aspect towards laser tunability. Among the investigated compounds, **PDI 10** and **PDI 7** have shown the best ASE threshold performance at low doping rates in the film, and more significantly at high concentrations, at which they show less aggregation than the others. Such behaviour is attributed to conformational (steric) effects, supported by NMR spectra. Also interesting is the observation of dual ASE in films doped at the lowest concentration with **PDI 11,** which constitutes a novel finding in PDIs. Overall, this study reveals the possibility to finely tune the ASE performance of 1,7 bay-substituted PDIs, in terms of emission wavelength and threshold, through proper selection of the type of substituents attached in the 1,7 bay-positions.

## Figures and Tables

**Table 1:** Optical properties of PDI derivates in liquid solution.

Compound	(1,6)/(1,7) ^a^	*λ*_ABS max_^b^ (nm)	*λ*_PL_^b^ (nm)	*ϕ*_PL_^c^
**PDI 6**	22/78	542	568	0.45
**PDI 7**	25/75	546	566	0.43
**PDI 8**	27/73	545	573	0.3
**PDI 9**	11/89	550	577	0.48
**PDI 10**	24/76	563	596	0.14
**PDI 11**	23/77	568	591	0.2

^a^ Determined by ^1^H NMR. ^b^ Optical properties measured in CHCl_3_. ^c^ Determined using *N,N′*-dihexylheptylperylene-3,4:9,10-tetracarboxidiimide as standard.

**Table 2:** Optical properties of PDI derivatives in PS-doped films.

Compound	PDI Conc. (μmol PDI/g PS) ^a^	*λ*_ABS_^b^ (nm)	*λ*_PL 0-0_^c^ (nm)	*α* (*λ*_Pump_) ^d^ (cm^−1^)	*λ*_ASE_ ^e^ (nm)	FHWM_ASE_ ^f^ (nm)	*I*_th_^ASE g^ (kW/cm^2^)
**PDI 6**	5.3	501, 538	560	643	596.7	16.4	87
	14.5	504, 538	563	1804	599.1	11.0	218
	29.5	505, 538	565	2620	598.6	8.2	581
**PDI 7**	4.9	510, 547	562	436	606.3	16.2	40
	14.9	510, 547	564	1069	609.7	13.9	40
	30.2	511, 547	567	2205	609.0	9.0	110
**PDI 8**	4.9	507, 543	560	372	599.8	14.2	127
	14.7	509, 543	564	1216	601.4	9.1	296
	30.7	510, 543	569	2306			
**PDI 9**	4.9	510, 547	565	429	604.7	19.5	50
	14.9	512, 547	575	1115	608.4	13.1	130
	30.2	512, 547	573	2191		8.8	780
**PDI 10**	4.9	518, 557	572	329	616.8	18.9	62
	14.9	518, 557	576	1123	620.4	15.5	49
	30.2	518, 557	578	1681	623.3	14.5	33
**PDI 11**	4.8	527, 569	582	305	596.9/631.8	10.6/13.8	167/129
	14.3	528, 569	585	993	632.7	7.7	243
	31.1	529, 569	587	2058			

^a^ *b*-PDI concentration in PS films. ^b^ Peak absorption wavelengths. ^c^ 0–0 peak photoluminiscence wavelength. ^d^ Absorption coefficient at *λ*_Pump_. ^e^ Amplified spontaneous emission (ASE) wavelength. ^f^ ASE linewidth defined as the full width at half maximum, FWHM, well above the threshold. ^g^ ASE threshold.

## Data Availability

The data supporting the findings of this manuscript are available from the corresponding authors upon reasonable request.

## Supplementary Materials

The following supporting information can be downloaded at: https://www.mdpi.com/article/10.3390/molecules28196776/s1, Figures S1 to S30: ^1^H and ^13^C NMR, UV-Vis, IR and MALDI-ToF mass spectra in solution for **PDI 6** to **PDI 11**. Figure S31: Thermogravimetric analysis of **PDI 6–11**. Figure S32: ASE intensity versus the number of pump pulses, for ASE operational lifetime investigation.
