# Supplementary material for: Effect of Different Substitutions at the 1,7-Bay Positions of Perylenediimide Dyes on Their Optical and Laser Properties
Source: Molecules. 2023 Sep 23;28(19):6776. doi: 10.3390/molecules28196776 (PMC10574636; doi:10.3390/molecules28196776)
Supplement: Supplementary file 1 [file molecules-28-06776-s001.zip › molecules-2540809-supplementary.pdf]

Effect of Different Substitutions at the 1,7-Bay Positions of Perylenediimide Dyes on Their Optical and Laser Properties

Nathalie Zink-Lorre [1,†], Manuel G. Ramírez [2,†], Sara Pla [1], Pedro G. Boj [3], José A. Quintana [3], José M. Villalvilla [4], Ángela Sastre-Santos [1], Fernando Fernández-Lázaro [1,*] and María A. Díaz-García [4,*]

1 Área de Química Orgánica, Instituto de Bioingeniería, Universidad Miguel Hernández de Elche, 03202 Elche, Spain; nzink@umh.es (N.Z.-L.); saplagar@hotmail.com (S.P.); asastre@umh.es (Á.S.-S.)
2 Instituto Universitario de Física Aplicada a las Ciencias y Tecnologías, Universidad de Alicante, 03080 Alicante, Spain; ramirez@mscloud.ua.es
3 Departamento de Óptica, Farmacología y Anatomía, andInstituto Universitario de Materiales (IUMA), Universidad de Alicante, 03080 Alicante, Spain; p.boj@ua.es (P.G.B.); ja.quintana@ua.es (J.A.Q.)
4 Departamento de Física Aplicada and Instituto Universitario de Materiales (IUMA), Universidad de Alicante, 03080 Alicante, Spain; jmvs@ua.es
* Correspondence: fdofdez@umh.es (F.F.-L.); maria.diaz@ua.es (M.A.D.G.)
† These authors contributed equally to this work.

***N*,*N'*-Di(ethylpropyl)-1,7(6)-diphenoxy-3,4:9,10-perylenetetracarboxydiimide** (**PDI 6**)

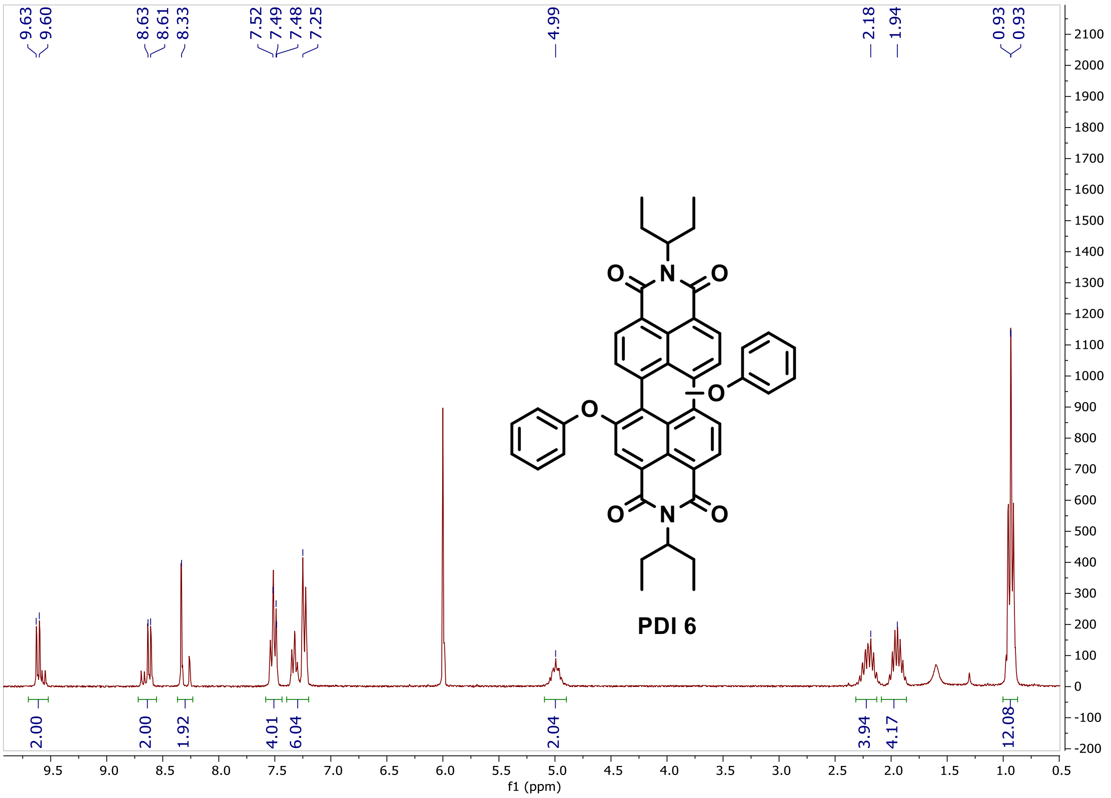

Figure S1: ^{1}H NMR spectrum of **PDI 6** in $C_2D_2Cl_4$.

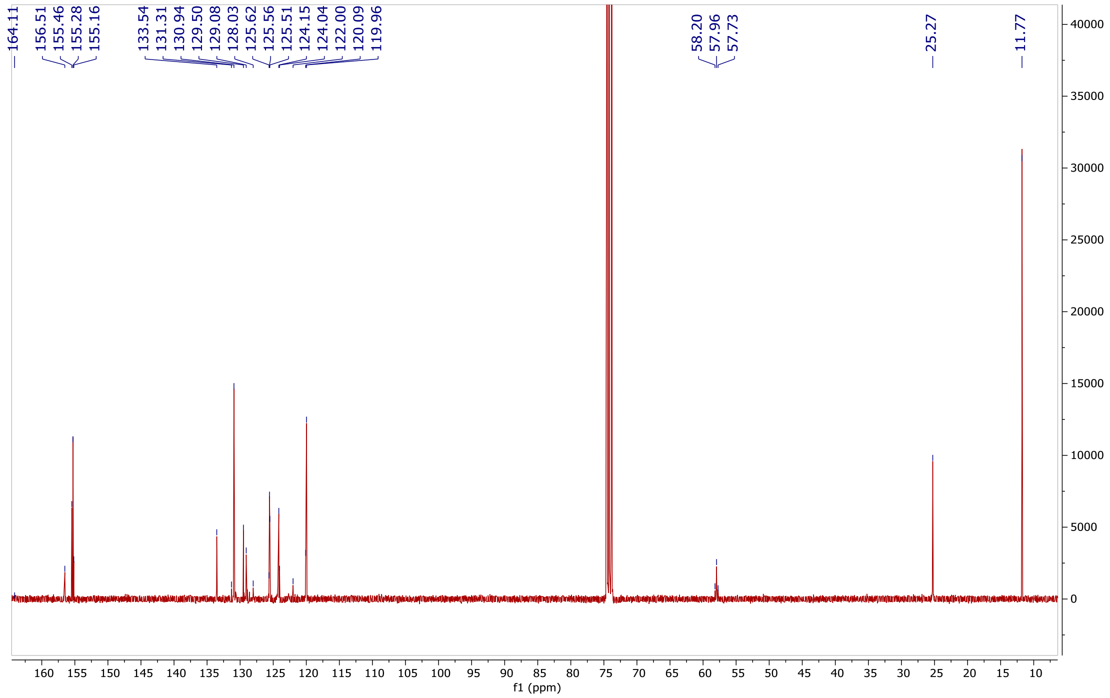

Figure S2: ^{13}C NMR spectrum of **PDI 6** in $C_2D_2Cl_4$.

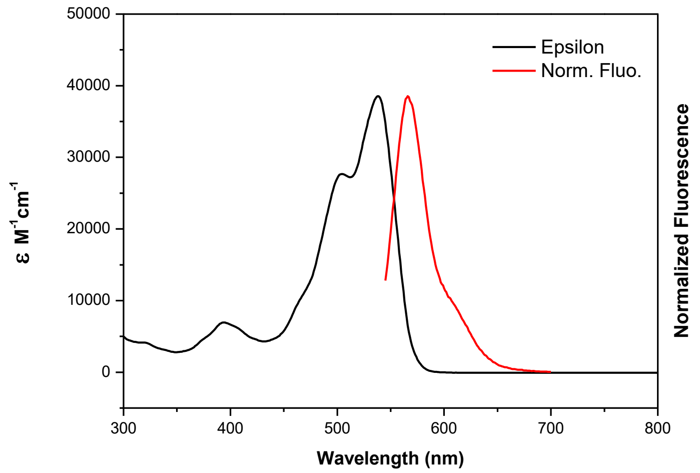

Figure S3: UV-vis and fluorescence (λ_{exc} = 542 nm) spectra of **PDI 6** in $CHCl_3$.

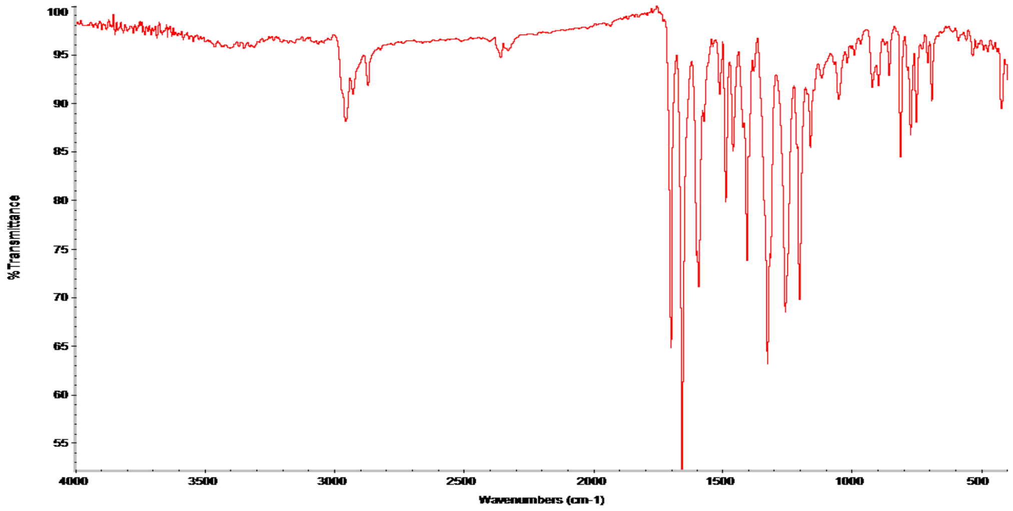

Figure S4: IR spectrum (KBr) of **PDI 6**.

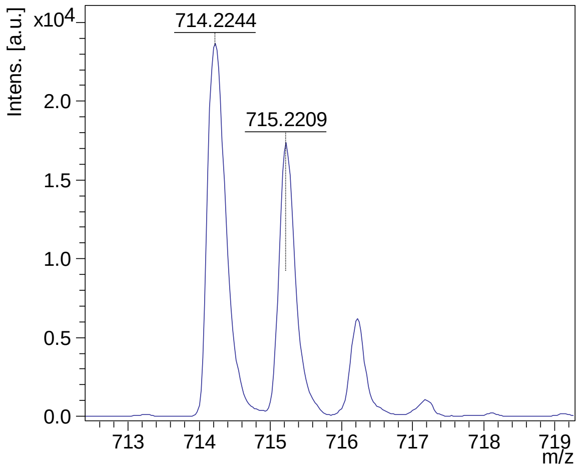

Figure S5: MALDI-ToF spectrum of **PDI-6**.

***N*,*N'*-Di(ethylpropyl)-1,7(6)-di(2,5-dimethylphenoxy)-3,4:9,10-perylenetetracarboxydiimide (PDI 7)**

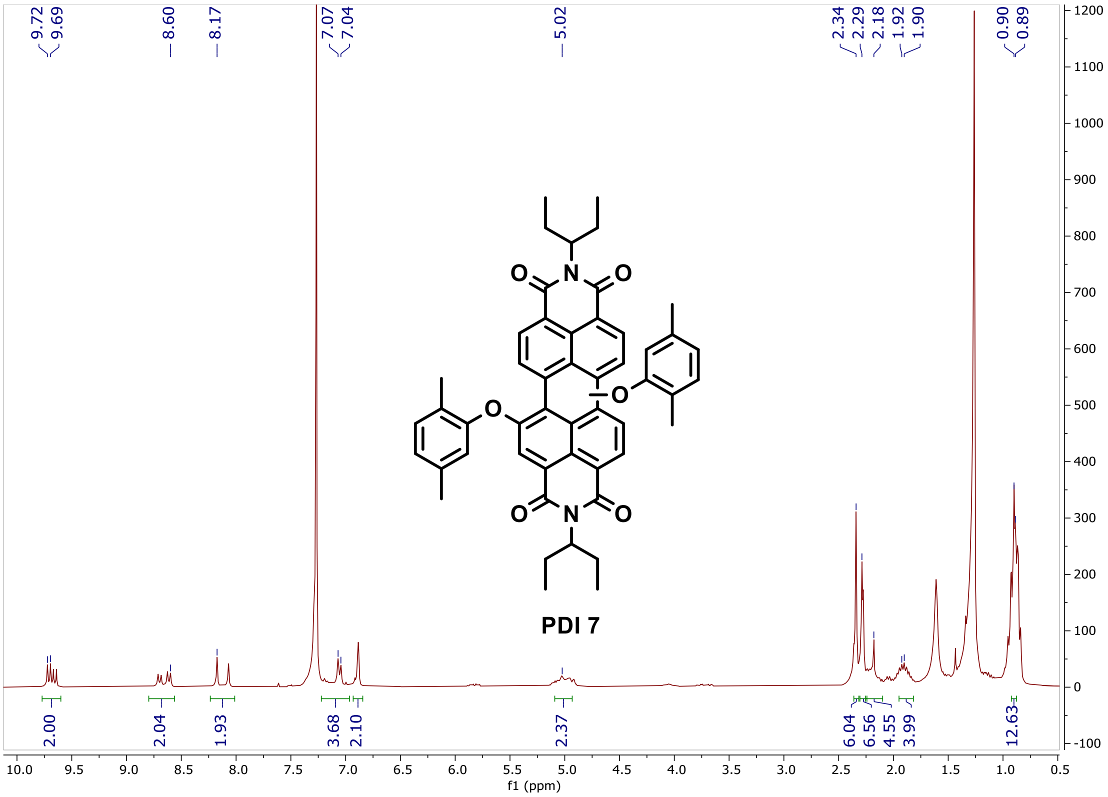

Figure S6: ^{1}H NMR spectrum of **PDI 7** in $CDCl_3$.

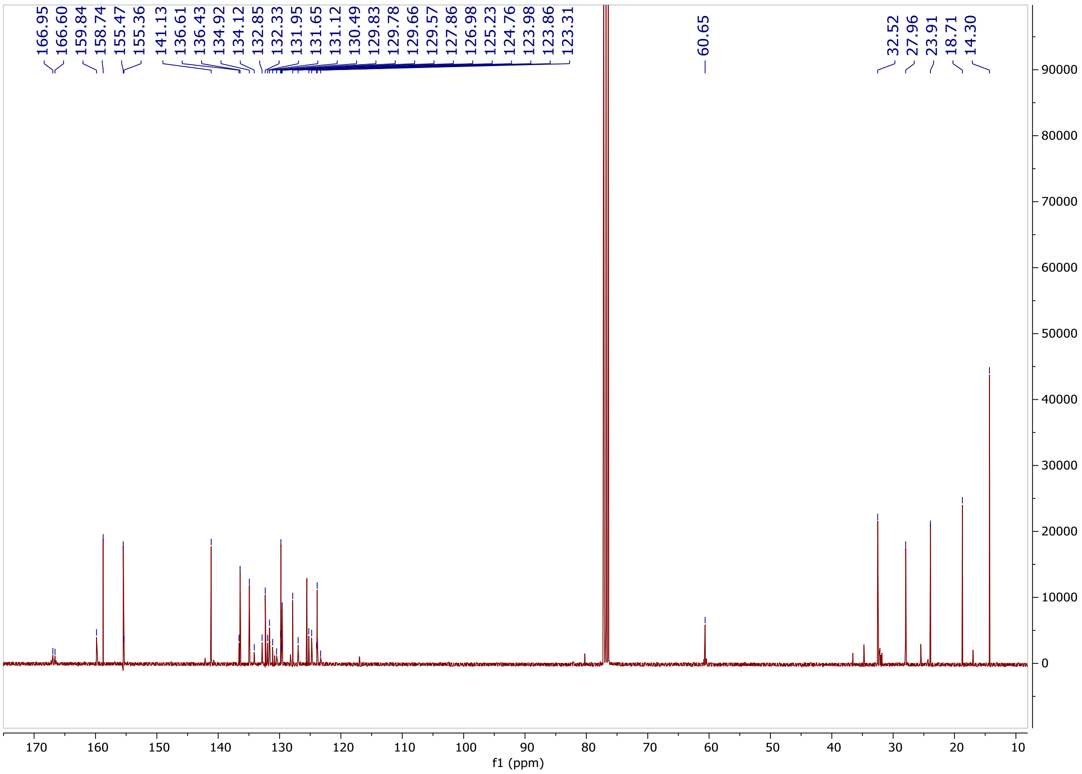

Figure S7: ^{13}C NMR spectrum of **PDI 7** in $C_2D_2Cl_4$.

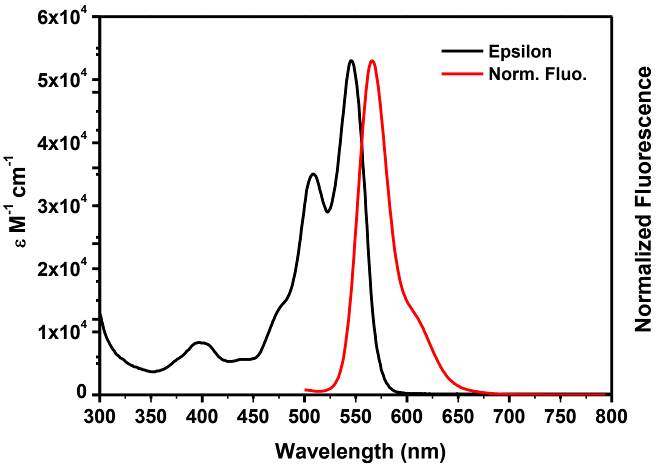

Figure S8: UV-vis and fluorescence (λ_{exc} = 546 nm) spectra of **PDI 7** in $CHCl_3$.

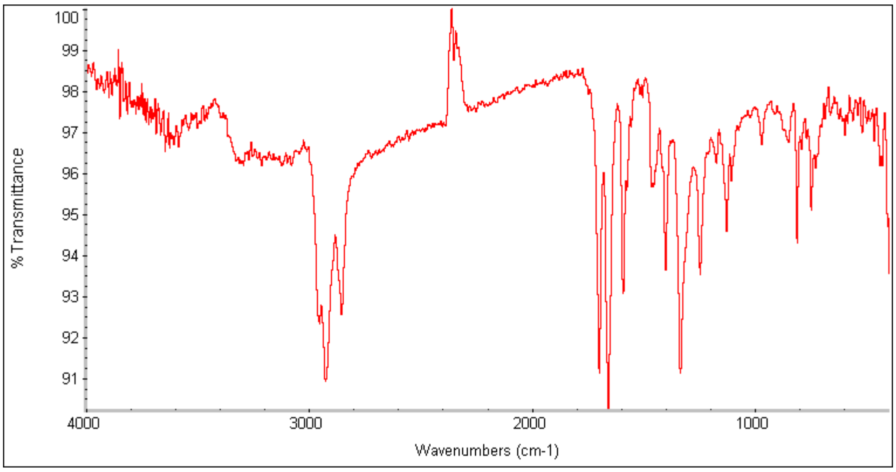

Figure S9: IR spectrum (KBr) of **PDI 7**.

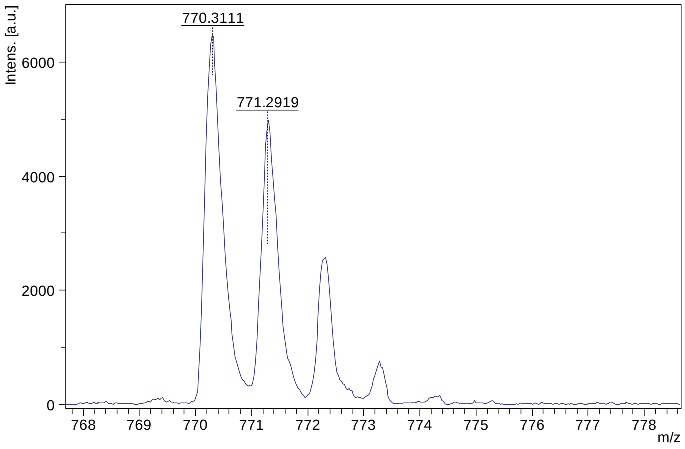

Figure S10: MALDI-ToF spectrum of **PDI-7**.

***N*,*N'*-Di(ethylpropyl)-1,7(6)-di(4-*t*-octylphenoxy)-3,4:9,10-perylenetetracarboxydiimide (PDI 8)**

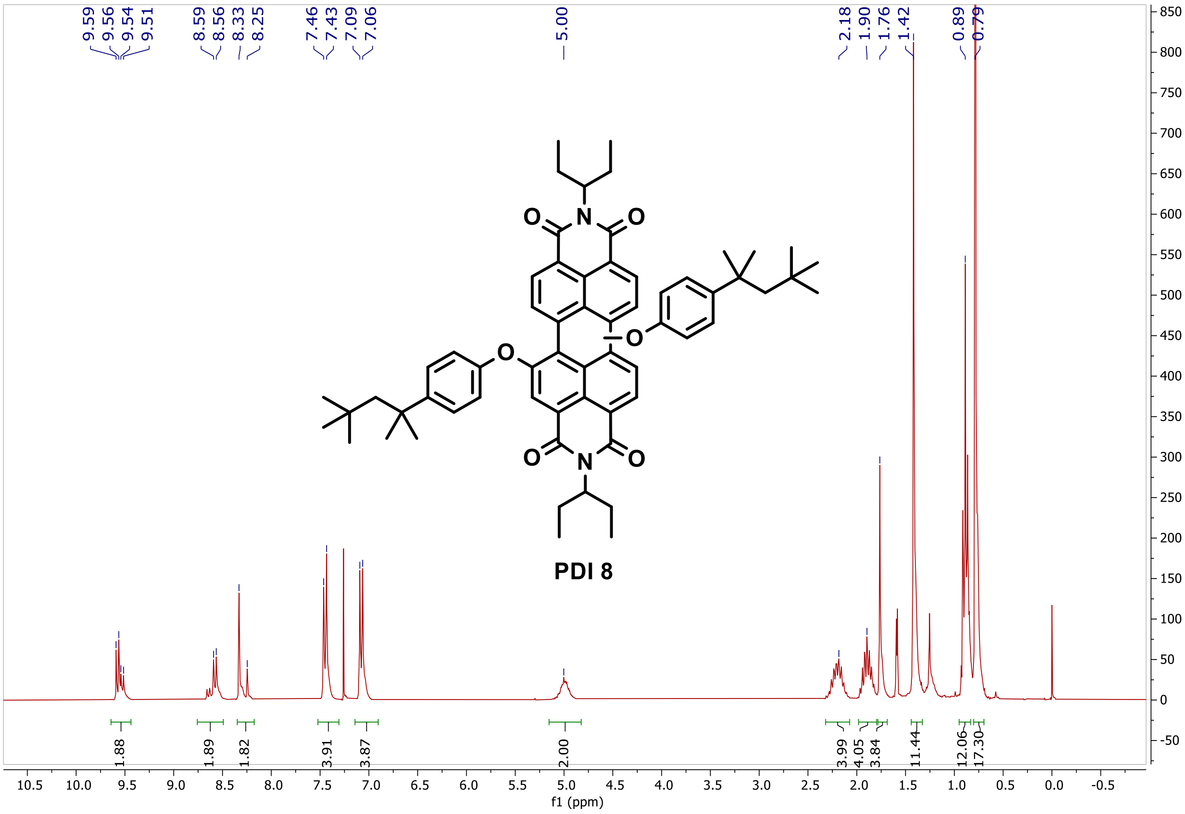

Figure S11: ^{1}H NMR spectrum of **PDI 8** in $CDCl_3$.

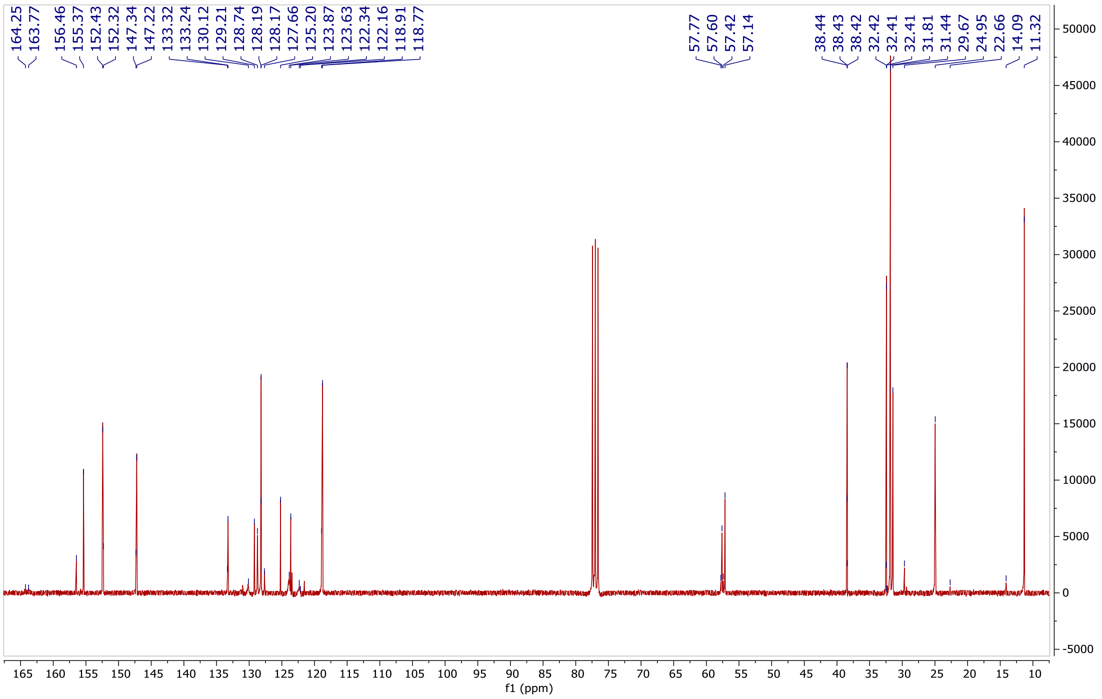

Figure S12: ^{13}C NMR spectrum of **PDI 8** in $CDCl_3$.

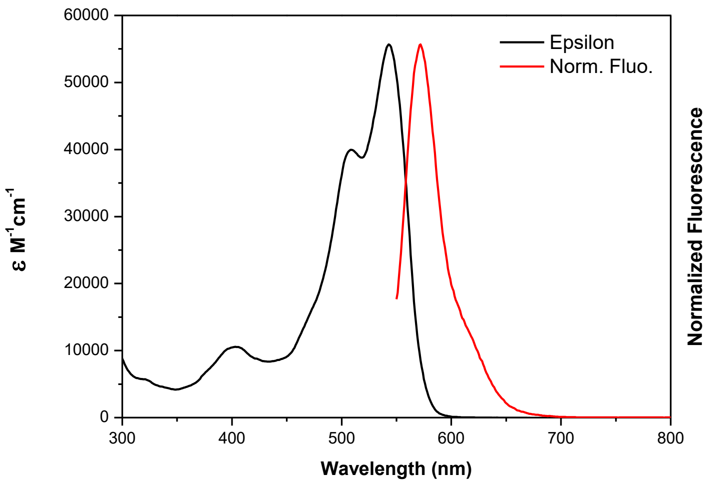

Figure S13: UV-vis and fluorescence (λ_{exc} = 545 nm) spectra of **PDI 8** in $CHCl_3$.

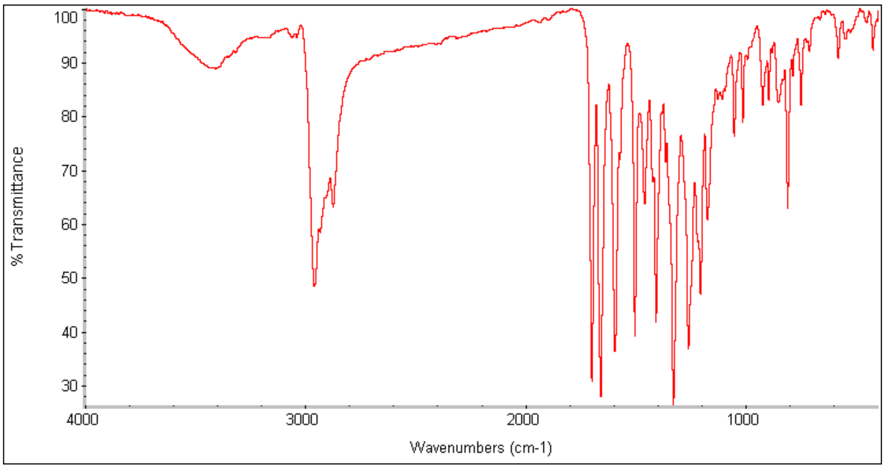

Figure S14: IR spectrum (KBr) of **PDI 8**.

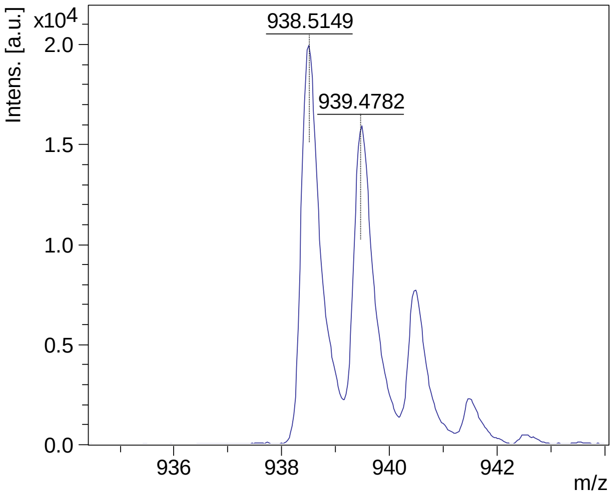

Figure S15: MALDI-ToF spectrum of **PDI-8**.

_N,N'_-Di(ethylpropyl)-1,7(6)-di(4-methoxiphenoxy)-3,4:9,10-perylenetetracarboxydiimide (PDI 9)

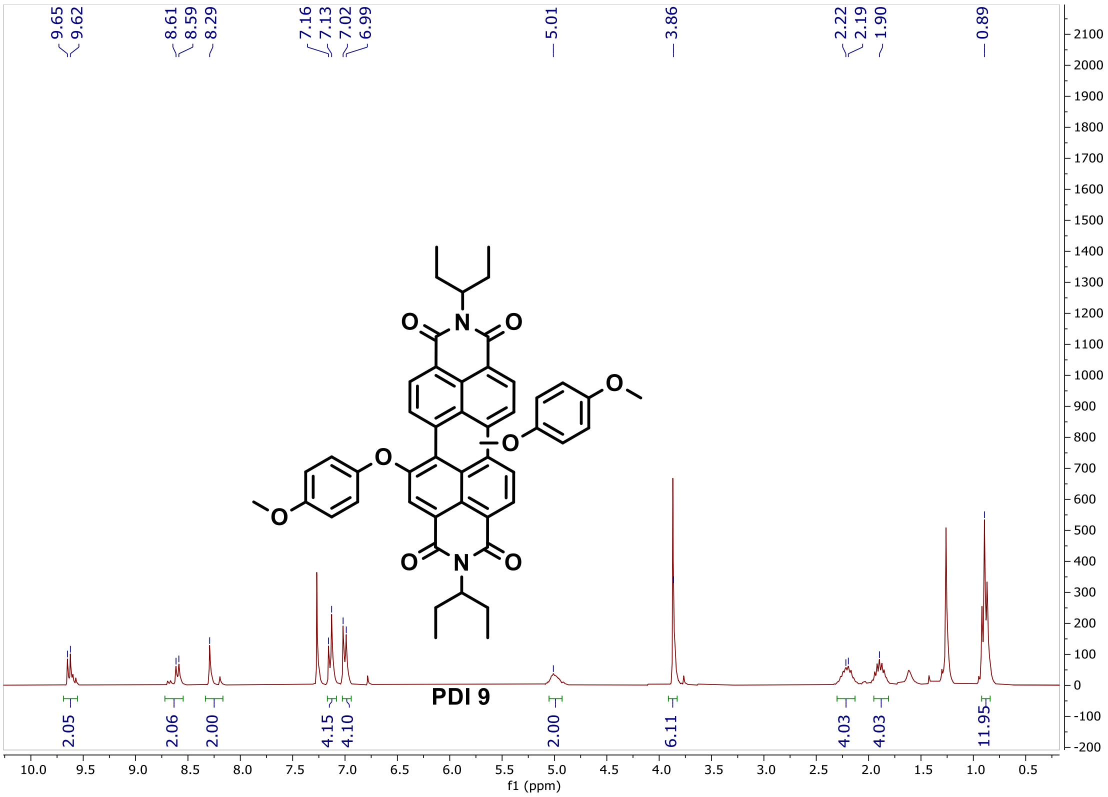

Figure S16: ^{1}H NMR spectrum of **PDI 9** in $CDCl_3$.

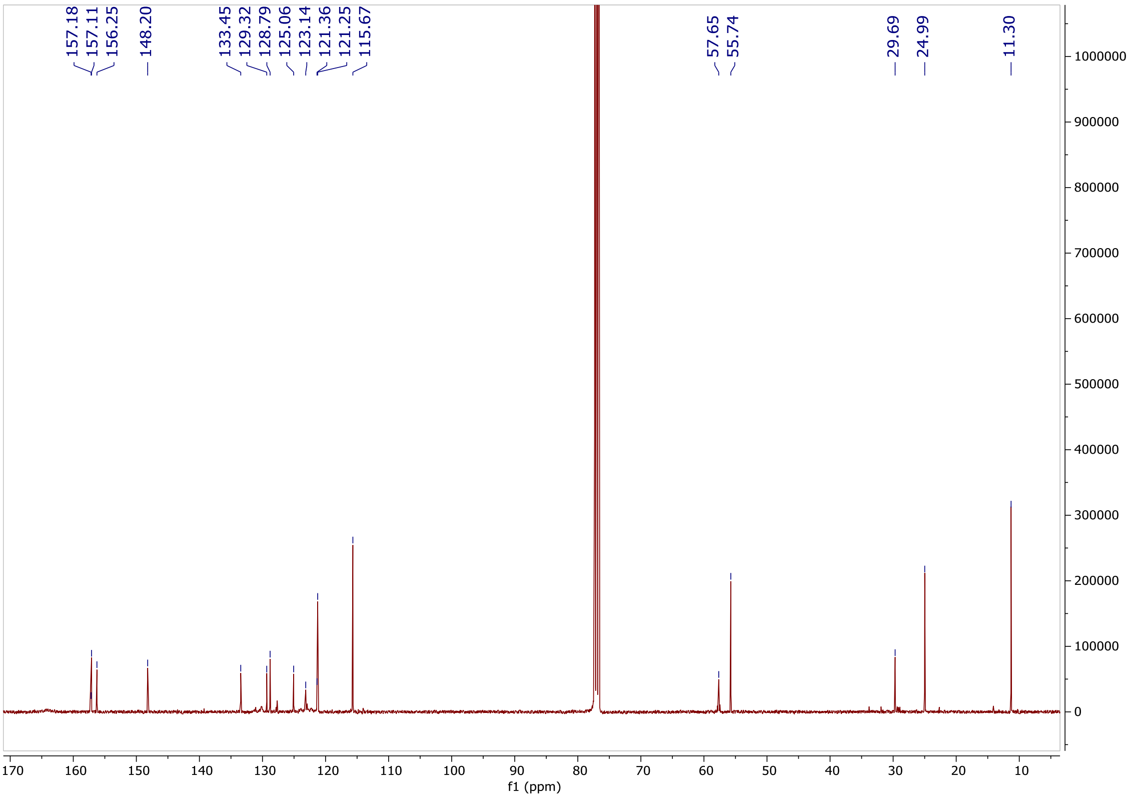

Figure S17: ^{13}C NMR spectrum of **PDI 9** in $CDCl_3$.

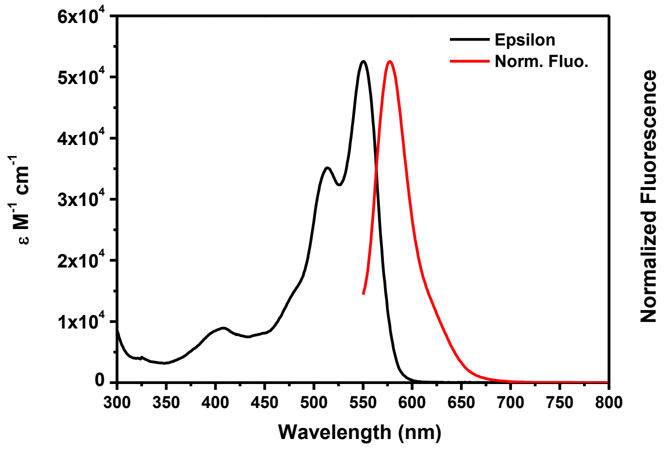

Figure S18: UV-vis and fluorescence (λ_{exc} = 550 nm) spectra of **PDI 9** in $CHCl_3$.

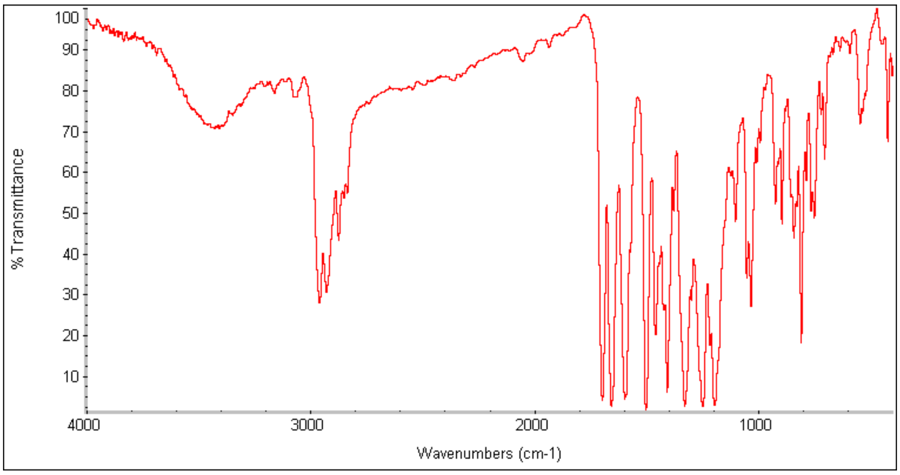

Figure S19: IR spectrum (KBr) of **PDI 9**.

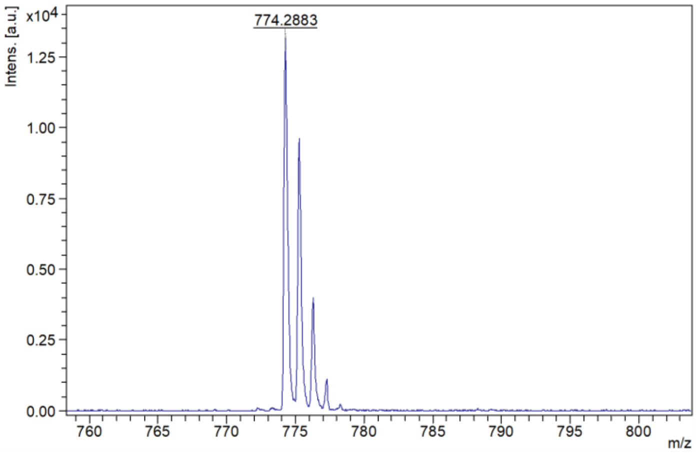

Figure S20: MALDI-ToF spectrum of **PDI-9**.

***N*,*N'*-Di(ethylpropyl)-1,7(6)-di[2,5-bis(1,1-dimethylbuthyl)-4-methylphenoxy]-3,4:9,10-perylenetetracarboxydiimide (PDI 10)**

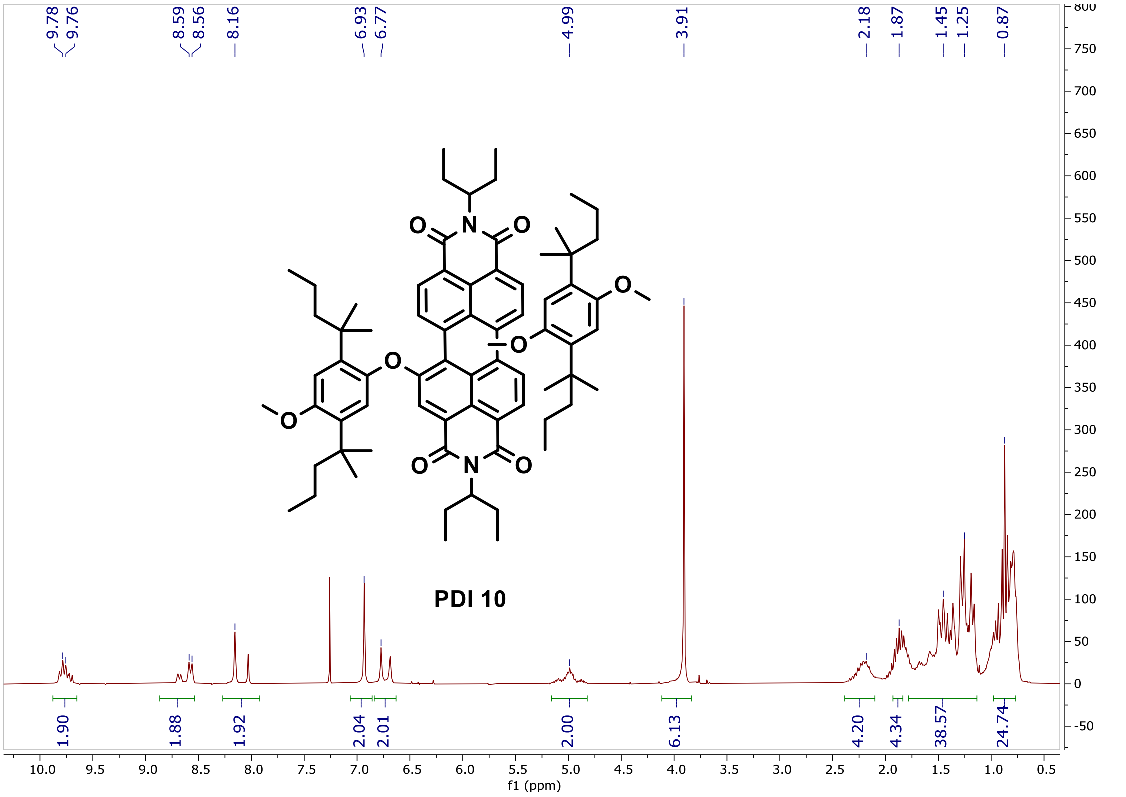

Figure S21: ^{1}H NMR spectrum of **PDI 10** in $CDCl_3$.

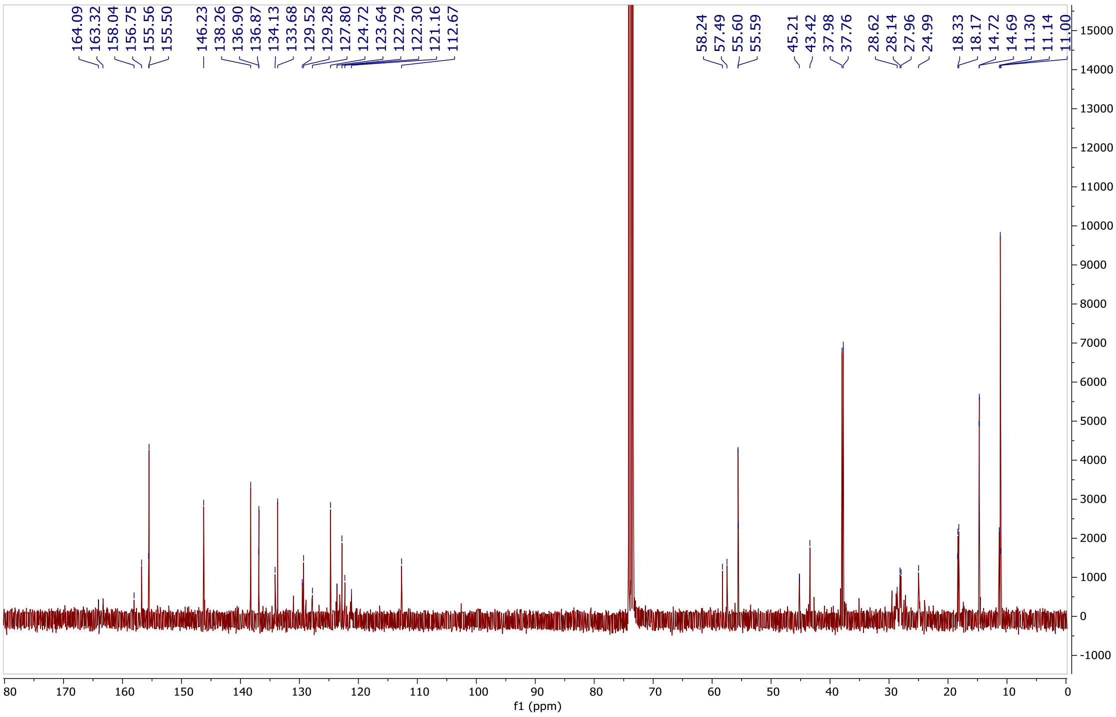

Figure S22: ^{13}C NMR spectrum of **PDI 10** in $C_2D_2Cl_4$.

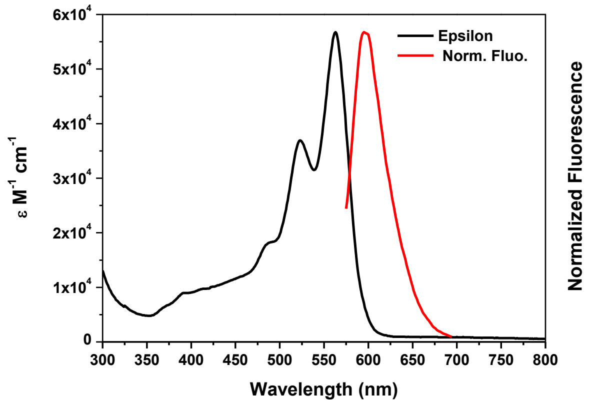

Figure S23: UV-vis and fluorescence (λ_{exc} = 563 nm) spectra of **PDI 10** in $CDCl_3$.

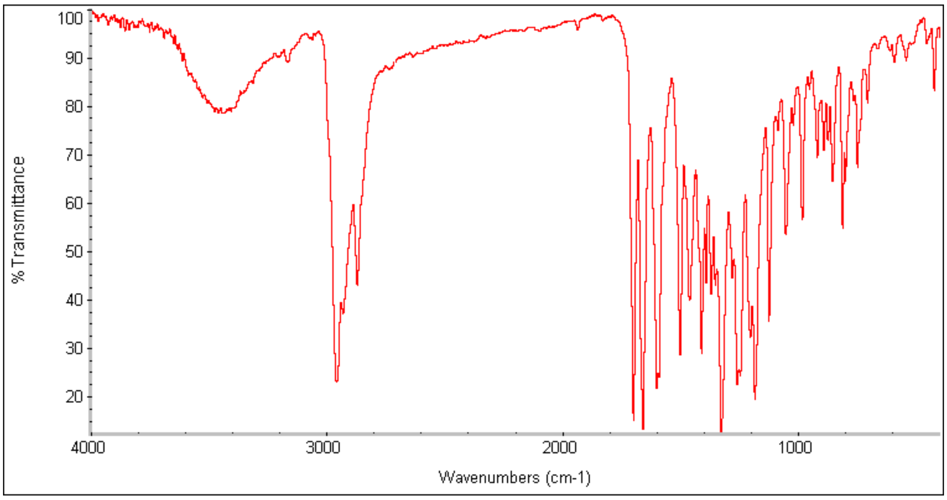

Figure S24: IR spectrum (KBr) of **PDI 10**.

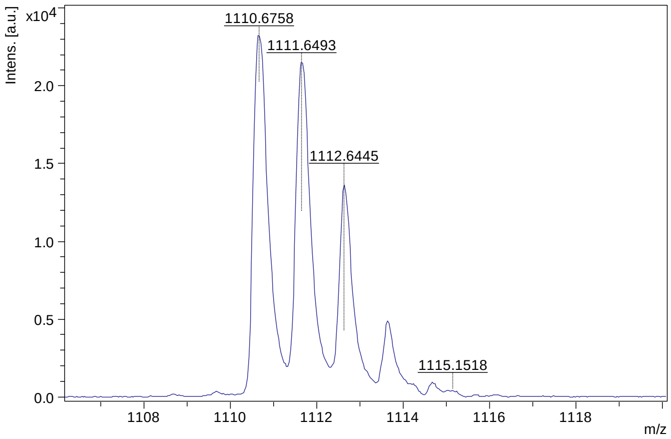

Figure S25: MALDI-ToF spectrum of **PDI-10**.

N,N'-Di(ethylpropyl)-1,7(6)-diethoxy-3,4:9,10-perylenetetracarboxydiimide (PDI 11)

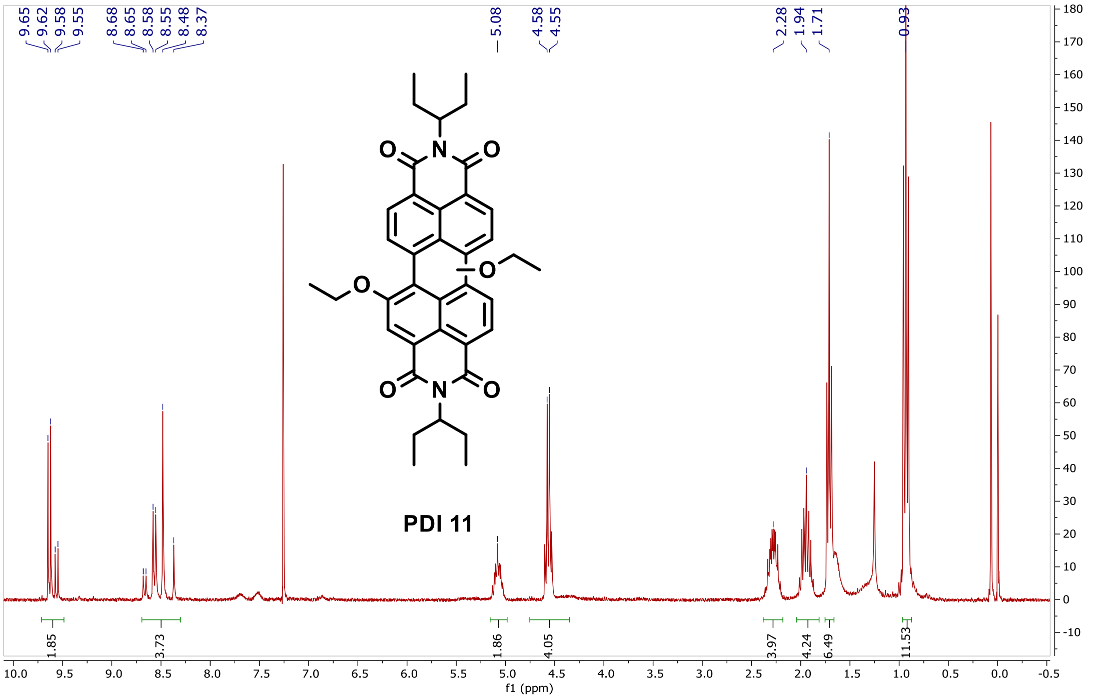

Figure S26: ^{1}H NMR spectrum of **PDI 11** in $CDCl_3$.

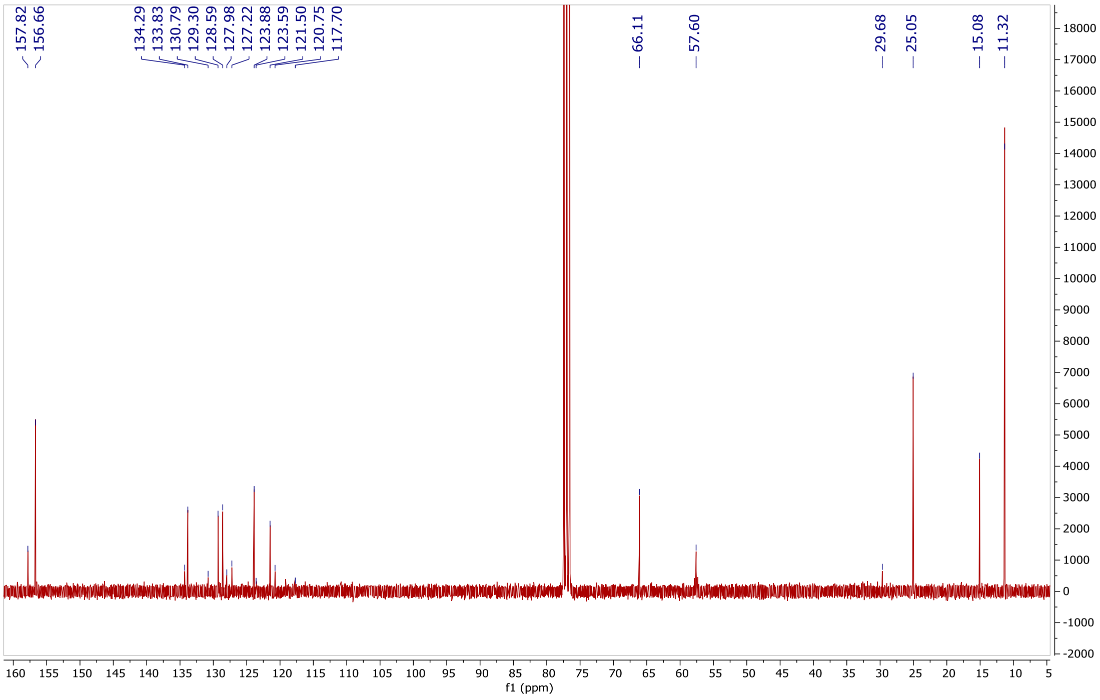

Figure S27: ^{13}C NMR spectrum of **PDI 11** in $CDCl_3$.

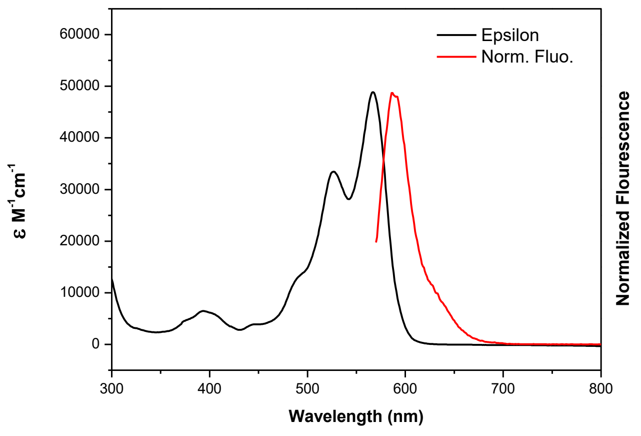

Figure S28: UV-vis and fluorescence (λ_{exc} = 568 nm) spectra of **PDI 11** in $CHCl_3$.

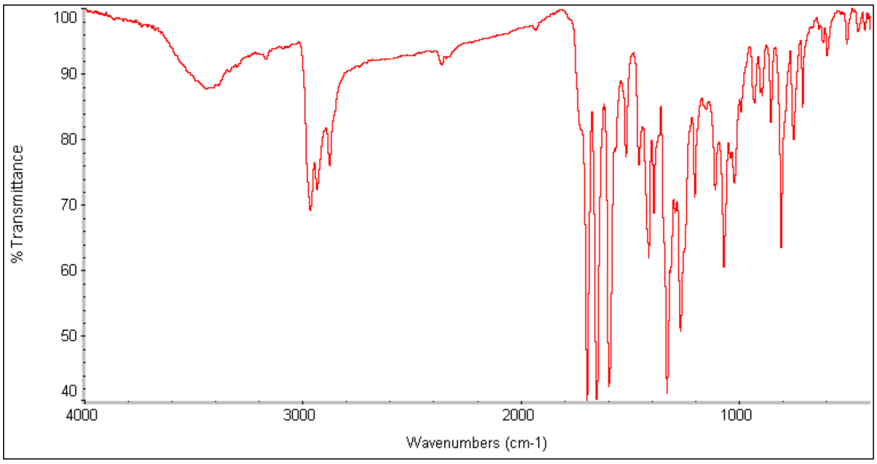

Figure S29: IR spectrum (KBr) of **PDI 11**.

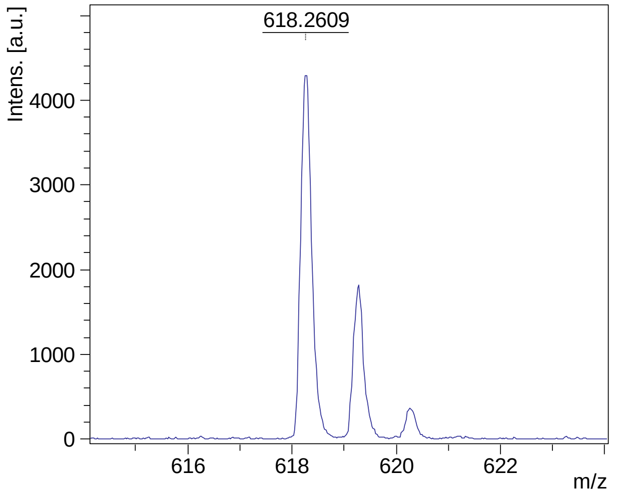

Figure S30: MALDI-ToF spectrum of **PDI-11**.

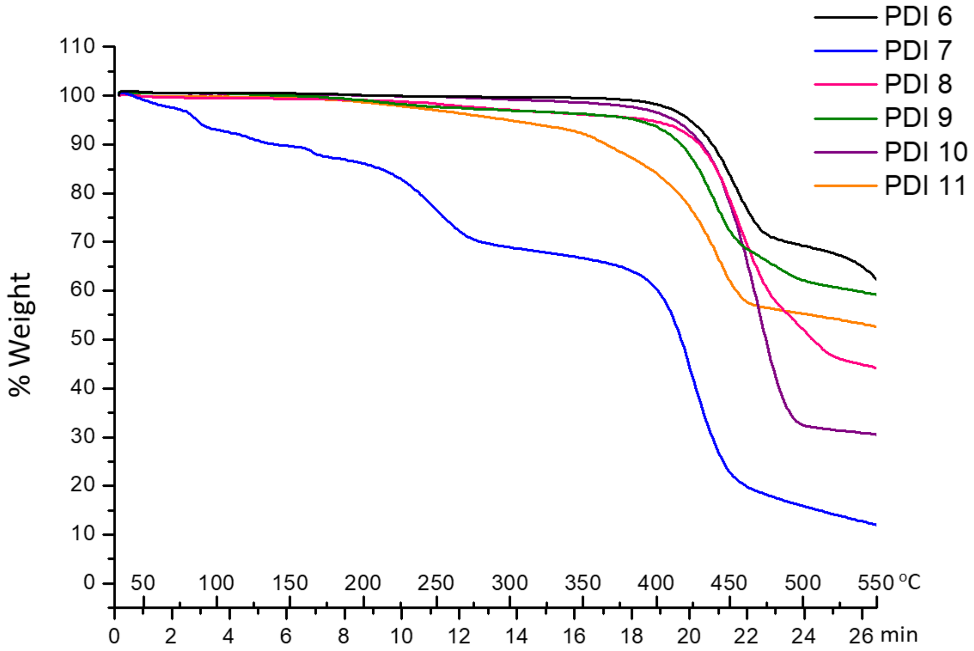

Figure S31: Thermogravimetric analysis of **PDI 6-11**.

a)

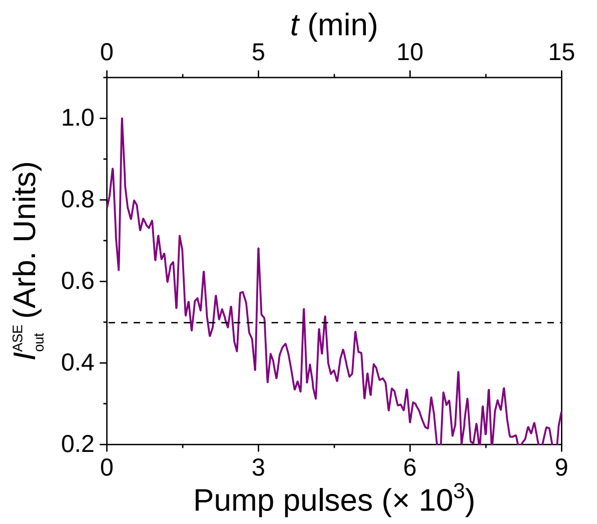

b)

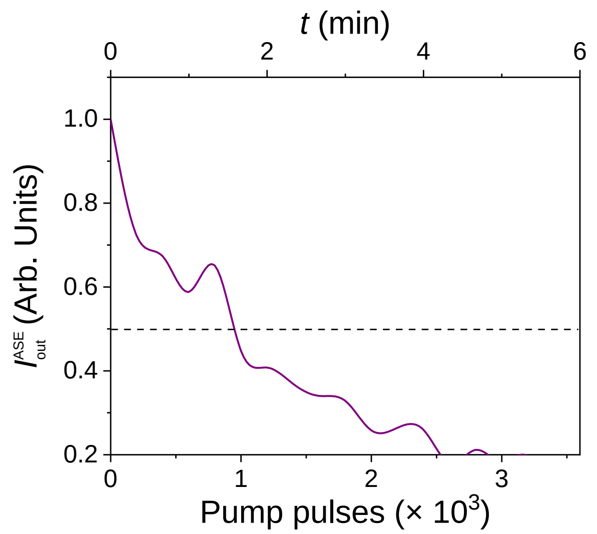

Figure S32: Normalized ASE intensity at the ASE wavelength, as a function of the number of pump pulses (10 ns, 10 Hz) and the time (botom and top axes, respectively), for PS films doped with 30.2 µmol PDI / g PS of **PDI 10**, under uninterrupted excitation at: (a) a moderate pump intensty of 70 kW cm^{-2} (around two times the ASE threshold); and (b) a high pump intensity of 2500 kW cm^{-2} (many times above threshold). The horizontal dashed line indicates the intensity at half of the initial value to determine the ASE half-life (around 2.8 × 10^3 and 0.8 × 10^3 pump pulses for (a) and (b), respectively)
